# The Optimal Age of Vaccination Against Dengue with an Age-Dependent Biting Rate with Application to Brazil

**DOI:** 10.1007/s11538-019-00690-1

**Published:** 2020-01-14

**Authors:** Sandra B. Maier, Eduardo Massad, Marcos Amaku, Marcelo N. Burattini, David Greenhalgh

**Affiliations:** 1 Department of Mathematics and Statistics, University of Strathclyde, Glasgow, G1 1XH U.K.; 2 LIM01-Hospital de Clínicas, Faculdade de Medicina, Universidade de São Paulo, São Paulo, SP Brazil; 3 Hospital São Paulo, Escola Paulista de Medicina, Universidade Federal de São Paulo, São Paulo, SP Brazil; 4 London School of Hygiene and Tropical Medicine, London, U.K.; 5 School of Applied Mathematics, Fundação Getulio Vargas, Rio de Janeiro, RJ Brazil

**Keywords:** Dengue, Vaccination, Optimal vaccination age, Age-structured model, Biting rate, Hospitalisation

## Abstract

In this paper we introduce a single serotype transmission model, including an age-dependent mosquito biting rate, to find the optimal vaccination age against dengue in Brazil with Dengvaxia. The optimal vaccination age and minimal lifetime expected risk of hospitalisation are found by adapting a method due to Hethcote (Math Biosci 89:29–52). Any number and combination of the four dengue serotypes DENv1–4 is considered. Successful vaccination against a serotype corresponds to a silent infection. The effects of antibody-dependent enhancement (ADE) and permanent cross-immunity after two heterologous infections are studied. ADE is assumed to imply risk-free primary infections, while permanent cross-immunity implies risk-free tertiary and quaternary infections. Data from trials of Dengvaxia indicate vaccine efficacy to be age and serostatus dependent and vaccination of seronegative individuals to induce an increased risk of hospitalisation. Some of the scenarios are therefore reconsidered taking these findings into account. The optimal vaccination age is compared to that achievable under the current age restriction of the vaccine. If vaccination is not considered to induce risk, optimal vaccination ages are very low. The assumption of ADE generally leads to a higher optimal vaccination age in this case. For a single serotype vaccination is not recommended in the case of ADE. Permanent cross-immunity results in a slightly lower optimal vaccination age. If vaccination induces a risk, the optimal vaccination ages are much higher, particularly for permanent cross-immunity. ADE has no effect on the optimal vaccination age when permanent cross-immunity is considered; otherwise, it leads to a slight increase in optimal vaccination age.

**Electronic supplementary material:**

The online version of this article (10.1007/s11538-019-00690-1) contains supplementary material, which is available to authorized users.

## Introduction

Dengue is considered the most important mosquito-borne viral disease of humans with half of the world’s population living in endemic areas and over 2 million dengue cases reported each year to the World Health Organization (2009, 2012). Due to the occurrence of asymptomatic infections and atypical clinical presentation dengue is in fact significantly under-reported so that the actual annual incidence is much higher (Gubler 2011); it has recently been estimated to be as high as 390 million cases of which approximately 100 million are symptomatic (Bhatt et al. 2013). These symptomatic cases of dengue fever (DF) are usually characterised by high fever accompanied by fatigue, rash, and headaches. If the disease manifests in one of its severe forms, i.e. dengue haemorrhagic fever (DHF) or dengue shock syndrome (DSS), symptoms can be plasma leakage and organ failure which can lead to death (Halstead 1980; Fredericks and Fernandez-Sesma 2014).

There are four distinct dengue virus serotypes DENv1–4 all of which are mainly spread by the *Aedes Aegypti* mosquito in Brazil and can cause any manifestation of dengue from an asymptomatic infection to severe dengue (SD). The coexistence of four serotypes entails the possibility of consecutive, heterologous infections which may be affected by interactions between serotypes and antibodies that were developed upon exposure to the different types. In fact, it is thought that a primary infection with any serotype leads to lifelong immunity specific to that type but protection against the other serotypes for a limited time only (Halstead 1980). Some studies have further shown that secondary infections cause 90–95% of cases of SD, with the remaining 5–10% being caused by primary infections, usually in infants between the ages of 6 and 12 months who have a low level of maternal antibodies (Leong et al. 2007; Halstead 2009; Jain and Chaturvedi 2010). Therefore, a consequence of the coexistence of several serotypes seems to be the enhancement of infection, particularly during secondary infections and during primary infections in infancy when maternal antibodies fall to low levels. This increase in infection severity is believed to be caused by a higher virulence which is in turn due to antibodies specific to the first serotype an individual was infected with or those passed on by the mother. These antibodies are cross-reactive with heterologous dengue types but non-neutralising and thus cause antibody-dependent enhancement (ADE) by binding on to the very similar dengue serotype and allowing the active virus entry into its target cells more easily (Halstead 2009; Jain and Chaturvedi 2010). Other observations regarding heterologous infections are that the sequence of serotypes with which individuals get infected influences the development of SD (Fried et al. 2010) and that two heterologous infections confer permanent cross-immunity (Gibbons et al. 2007; Anderson et al. 2013). Considering all of these complex interdependencies it is not surprising that instead of vaccines mainly vector control strategies were used to prevent the transmission of dengue in the past.

The development of a dengue vaccine was a complicated and lengthy process; however, in December 2015 after 20 years of development Sanofi Pasteur licensed Dengvaxia, the first vaccine against dengue (Sanofi Pasteur Press Release 2015). Since then it has been licensed for the use in individuals between the ages of either 9 and 45 or 9 and 60 years in more than ten countries including Brazil (Sanofi Pasteur Press Release 2016). Even before the licensure of Dengvaxia mathematical models had been used to predict the impact vaccination could have on the spread of dengue, and considering the complicated interdependencies like ADE and short-term cross-protection there is unsurprisingly some dispute about the effects of vaccination. While there is an overall agreement that vaccination could reduce DF cases significantly (Coudeville and Garnett 2012; Knipl and Moghadas 2015), there are indications that vaccination in the presence of ADE could lead to more SD cases (Knipl and Moghadas 2015). Ferguson et al. (2016) draw the conclusion that the transmission setting plays an important role in whether dengue vaccine will be beneficial or harmful by making the assumption that vaccination acts as a silent natural infection and by using a mathematical transmission model to show that in low-transmission settings vaccination may lead to more SD cases, whereas in high-transmission settings vaccination will be favourable both for the population as a whole and for the vaccinated individual. It can therefore be said that the phenomenon of ADE in dengue infections poses a great challenge for the development of vaccines since it makes it necessary to achieve a successful immune response to all four serotypes in order to prevent the creation of enhancing antibodies (Stephenson 2005). Dengvaxia, the only available vaccine at the moment, has been shown to be at least partially effective against all dengue serotypes in several Phase III trials; however, it has been found to have different efficacies for each of the serotypes and these efficacies further seem to depend on the age at vaccination and the serostatus of the vaccine recipient (Capeding et al. 2014; Hadinegoro et al. 2015). Since the licensing of Dengvaxia concerns about its application have been raised; in particular its use in seronegative recipients has been questioned since in this group an increase in the risk of acquiring SD in a subsequent natural infection has been observed (Aguiar et al. 2016; Halstead and Russell 2016; SAGE/World Health Organization 2016). This observation seems to be in agreement with the findings of Ferguson et al. (2016) pointing towards vaccination causing ADE in the first natural infection if seronegative individuals are vaccinated.

While recently much attention has been given to the possible effects of vaccination and to the optimisation of vaccination strategies in order to employ the most cost-effective strategy of vaccination or to achieve herd immunity and eradication of the disease (Billings et al. 2008; Durham et al. 2013) the age at which vaccination should ideally take place has rarely been considered. However, mathematical modelling has in the past been used to find optimal vaccination ages for other infectious diseases such as rubella and measles (Hethcote 1988; Anderson and May 1983) and we employ a method due to Hethcote (1988) to do the same for dengue. The existence of four dengue serotypes requires us to extend this method to take account of any number of serotypes existing in one endemic area, and we further take account of the survival probability of humans which Hethcote (1988) neglected. Additionally, while in many dengue transmission models the mosquito biting rate and the human mortality rate are assumed constant, we want to model more realistic transmission dynamics by assuming an age-dependent mosquito biting rate and a step death function which is more realistic for countries like Brazil. In this paper our objective is therefore to find optimal vaccination ages for dengue when the aim of vaccination is to reduce the risk of hospitalisation due to SD. While considering a single serotype transmission model to achieve this we still take account of the possible coexistence of multiple serotypes in an endemic area and the assumptions relating to their interactions by utilising a risk function to incorporate them into the model. Additionally, we investigate the optimal vaccination age when vaccination of seronegative individuals can have negative effects.

## Vaccination Model

In order to find optimal vaccination ages for dengue we model the transmission of the virus in the presence of vaccination. To do this we assume independent transmission of the four distinct dengue serotypes which allows us to use a single serotype transmission model where some of the parameters are interchangeable to describe any one of the serotypes. Considering observations regarding interactions between the serotypes such as short-term cross-immunity and ADE this is only an approximation of the real dynamics. However, since these interactions are observed only in the short term the model can be considered to be a reasonable approximation. We further incorporate a three-dose vaccination strategy based on the restrictions under which Dengvaxia is licensed in Brazil by requiring a set of matching conditions to be met in addition to the initial conditions of the transmission model. These matching conditions include parameters that are serotype specific to allow for different vaccine efficacies for each serotype and at different vaccination ages.

### Single Serotype Transmission Model

The Ross–MacDonald model is a common way to describe vector-borne infections such as dengue (Esteva and Vargas 1998; Garba et al. 2008), and the model we use is in fact of this type with the relevant modification of considering age density functions rather than total numbers for the human population.

We assume that humans potentially progress through four different stages in their life. Every human is born passively immune due to maternal antibodies, once these antibodies decline the individual becomes susceptible to the virus so that an adequate contact with the virus leads to infection with dengue from which an individual eventually recovers. Once humans recover they are immune to the serotype that they were infected with for the remainder of their life. By taking the loss of passive immunity to be given by an age-dependent function *C*(*a*) which is estimated for each of the four dengue serotypes individually using data on the decline of maternal antibodies given by van Panhuis et al. (2011) and by taking the death rate to be the same for both passively immune and susceptible individuals we can in fact consider one compartment of unaffected which comprises both the passively immune and the susceptible. Therefore, the age densities $U_H(a,t)$, $I_H(a,t)$, and $R_H(a,t)$ of ‘unaffected’, ‘infected’, and ‘recovered’ humans at time *t* are modelled. The age densities for passively immune and susceptible individuals are then given by $(1-C(a))U_H(a,t)$ and $C(a)U_H(a,t)$, respectively, and the age density for the entire human population is $N_H(a,t) = U_H(a,t) + I_H(a,t) + R_H(a,t)$.

As an approximation we consider the total population size $N_H = N_H(t) = \int _0^{\infty }N_H(a,t)\hbox {d}a$ to be constant over time. The death rate for all compartments is given by a step death function, i.e. we assume that all humans die at age *L*. The age-dependent natural mortality rate $\mu _H(a)$ and the survival probability $\pi (a)$ for the human population are therefore given by

1 $$\begin{aligned} \mu _H(a)&= {\left\{ \begin{array}{ll} 0, &{} 0 \le a< L \\ \infty , &{} L \le a < \infty , \end{array}\right. } \end{aligned}$$

2 $$\begin{aligned} \pi (a)&= {\left\{ \begin{array}{ll} 1, &{} 0 \le a< L \\ 0, &{} L \le a < \infty . \end{array}\right. } \end{aligned}$$

These assumptions lead to the step function equilibrium human age distribution

3 $$\begin{aligned} N(a) = N(a,t) = \frac{N_H}{L}\pi (a). \end{aligned}$$

Note that a step death function is a more realistic representation than a constant death rate which is commonly used in these types of models. We shall take the death rate to be the same in all compartments corresponding to the assumption that dengue does not cause any additional deaths. This is reasonable since only 25,000 deaths are caused by dengue out of 390 million infections annually (Bhatt et al. 2013; Gibbons and Vaughn 2002). For the progression from unaffected to infected we assume that humans are bitten by mosquitoes at a rate *q*(*a*) depending on their age according to mosquito biting data (Massad 2015). Further if a susceptible human is bitten by an infectious mosquito, the probability of the human becoming infected with dengue is denoted by *b*. Using these parameters and functions, and noting that the number of infectious mosquitoes is denoted by $I_M(t)$, the force of infection for the human population is

4 $$\begin{aligned} \lambda _H(a,t) = bq(a)I_M(t)\frac{1}{N_H}. \end{aligned}$$

Once an individual is infected they recover from dengue at a constant rate $\gamma _H$, and once recovered stay immune until they die. The set of differential equations for the human population is therefore given by Eq. (6).

For the mosquito population we allow progression through three compartments, with all mosquitoes being born susceptible. Susceptible mosquitoes become exposed upon an adequate contact with dengue and infectious after a latency period $\tau$. Infectious mosquitoes do not recover from dengue but leave the compartment only once they die. The larval stage is omitted in this model since we are interested in the vaccination age of humans, so that new mosquitoes are directly recruited into the susceptible compartment at a constant rate $\mu _M$. Exposure to the virus, i.e. a susceptible mosquito biting an infected human, causes transmission of the virus with probability *c*. We have already noted that an age-dependent biting rate *q*(*a*) is assumed and that the age density of infected humans is given by $I_H(a,t)$ so that adequate contacts take place at a rate $\int _0^{\infty }q(a)I_H(a,t)\frac{1}{N_H}\hbox {d}a$ and the force of infection in the mosquito population is

5 $$\begin{aligned} \lambda _M(t) = c\int _0^{\infty }q(a)\frac{I_H(a,t)}{N_H}\hbox {d}a. \end{aligned}$$

The size of the mosquito population $N_M$ is assumed constant, and the death rate for all mosquitoes is given by $\mu _M$ independent of the compartment they are in. For the mosquito population the set of differential equations is then given by Eq. (7).

The single serotype transmission model with a step death function for the human population and an age-dependent biting rate is

6 $$\begin{aligned}&\frac{\partial U_H}{\partial a}+\frac{\partial U_H}{\partial t} = -\lambda _H(a,t)C(a)U_H(a,t) - \mu _H(a)U_H(a,t), \nonumber \\&\frac{\partial I_H}{\partial a}+\frac{\partial I_H}{\partial t} = \lambda _H(a,t)C(a)U_H(a,t) - (\mu _H(a) + \gamma _H)I_H(a,t), \nonumber \\&\frac{\partial R_H}{\partial a}+\frac{\partial R_H}{\partial t} = \gamma _HI_H(a,t) - \mu _H(a)R_H(a,t), \nonumber \\&\frac{\partial N_H}{\partial a}+\frac{\partial N_H}{\partial t} = -\mu _H(a)N_H(a,t), \nonumber \\&N_H(a,t) = U_H(a,t) + I_H(a,t) + R_H(a,t), \end{aligned}$$

7 $$\begin{aligned}&\frac{\hbox {d}S_M}{\hbox {d}t} = -\lambda _M(t)S_M(t) - \mu _MS_M(t)+ \mu _MN_M, \nonumber \\&\frac{\hbox {d}L_M}{\hbox {d}t} = \lambda _M(t)S_M(t) - e^{-\mu _M \tau } \lambda _M(t-\tau )S_M(t-\tau ) - \mu _ML_M(t), \nonumber \\&\frac{\hbox {d}I_M}{\hbox {d}t} = e^{-\mu _M \tau } \lambda _M(t-\tau )S_M(t-\tau ) - \mu _MI_M(t), \nonumber \\&N_M(t) = S_M(t) + L_M(t) + I_M(t), \end{aligned}$$

where the initial conditions are given by

$$\begin{aligned} \begin{array}{ccc} U_H(a,0) =U_{H,0}(a), &{} I_H(a,t) = I_{H,0}(a) \ \hbox {for} \ \tau \in [-\tau ,0], &{} R_H(a,0) = R_{H,0}(a), \\ U_H(0,t) =\frac{N_H}{L} \ , &{} I_H(0,t)=0, \qquad \qquad \qquad \qquad \quad \ \ &{} R_H(0,t)=0, \qquad \quad \ \ \\ \end{array} \end{aligned}$$

for the human population, and by

$$\begin{aligned} \begin{array}{ccc} S_M(t) = S_{M,0} \ \hbox {for} \ \tau \in [-\tau ,0],&L_M(0)=L_{M,0},&I_M(0)=I_{M,0}, \end{array} \end{aligned}$$

for the mosquito population, and the parameters and age-dependent functions that are used are summarised in Table 1.

**Table 1:** Description parameters and age-dependent rates used in the model

Parameter	Significance
*q*(*a*)	Total rate per unit time at which a single mosquito bites humans of age *a* given by Eq. (11)
*b*	Probability per bite that an initially susceptible human bitten by an infected mosquito becomes infected
*c*	Probability per bite that an initially susceptible mosquito biting an infected human becomes infected
$N_H$	Total number of humans
*L*	Expected lifetime of humans in Brazil
$\mu _H(a)$	Step death rate depending on *L*
$\gamma _H$	Per capita recovery rate of humans
$N_M$	Total number of mosquitoes
$\mu _M$	Natural per capita death rate of mosquitoes
$\tau$	Incubation period in mosquitoes (the extrinsic incubation period)
$A_i$ ($i=1,2,3$)	Vaccination age for each of the three vaccination stages
$V_i$ ($i=1,2,3$)	Vaccinated proportion of the population for each vaccination age

### Vaccination Strategy

So far the model described in Eqs. (6) and (7) does not include any vaccination one way of incorporating a vaccination strategy in the model is to add matching conditions for each vaccination dose. Using the function *C*(*a*) as the seroconversion rate as was done by Hethcote (1988) and assuming a fraction $V_i$ of the population is vaccinated at age $A_i$ the probability of becoming immune due to vaccination at age $A_i$ is given by $V_iC(A_i)$. Consequently this means the probability of staying unaffected at age $A_i$ is $1-V_iC(A_i)$ so that there is a jump decrease in the age density of susceptible humans which leads to the matching condition

8 $$\begin{aligned} \lim _{a \rightarrow A_i^+} U_H(a,t) = (1-V_iC(A_i))\lim _{a \rightarrow A_i^-} U_H(a,t) \end{aligned}$$

for each vaccination dose *i*. Note that the vaccinated fraction $V_i$ can be utilised to incorporate the vaccine efficacy.

Combining the single serotype transmission model with the matching conditions obtained by including vaccination it is now possible to model each of the four dengue serotypes. The variation in the serotypes can be modelled by using different seroconversion rates *C*(*a*), as well as by substituting different values for the vaccine efficacy and therefore different fractions $V_i$. Note that successful vaccination against a serotype is essentially a silent infection with that serotype.

## Basic Reproduction Number $R_0$

The basic reproduction number $R_0$ is considered to be one of the most important quantities in epidemiology and is defined as the number of secondary infections produced from a single infected individual in a naive population, i.e. an entirely susceptible population (Diekmann et al. 1990). It is therefore possible to obtain $R_0$ for the transmission model described in Eqs. (6) and (7) intuitively. The basic reproduction number for our model is given by

9 $$\begin{aligned} R_0 = \frac{mbce^{-\mu _M\tau }}{\mu _M}\frac{1}{L}\int _0^{\infty }q(a)C(a)\int _a^{\infty }q(s)e^{-\gamma _H\left( s-a\right) }\pi (s)\hbox {d}s\hbox {d}a \end{aligned}$$

where $m = N_M/N_H$ is the ratio of the number of mosquitoes to humans as outlined in Supplementary Appendix A.1.

Since our aim is to consider the coexistence of several serotypes we require values of $R_0$ specific to each of the serotypes. In the literature there is a wide range of parameter values assumed for general dengue models (Feng and Velasco-Hernández 1997; Coutinho et al. 2006) that could be used to compute $R_0$ as given in Eq. (9). Instead of determining all parameters for each of the serotypes we want to estimate serotype-specific $R_0$ from the initial phase of an outbreak as has been done for dengue before (Massad et al. 2010, 2001; Favier et al. 2006). This is a more robust method as it does not depend on estimating a large number of parameters. In order to do this an approximation of the basic reproduction number needs to be obtained by linearising the model equations and expressing $R_0$ in terms of $\lambda$ the initial growth rate of an infection. The approximate expression

10 $$\begin{aligned} R_0 \approx \frac{\lambda +\mu _M}{\mu _M} e^{\lambda \tau } \frac{\int _0^{\infty }q(a)C(a)\int _a^{\infty }q(s)e^{-\gamma _H\left( s-a\right) }\pi (s)\hbox {d}s\hbox {d}a}{\int _0^{\infty }q(a)C(a)\int _a^{\infty }q(s)e^{-\left( \lambda +\gamma _H\right) \left( s-a\right) }\pi (s)\hbox {d}s\hbox {d}a} \end{aligned}$$

can be found for the basic reproduction number as outlined in Supplementary Appendix A.2.

**Fig. 1:**
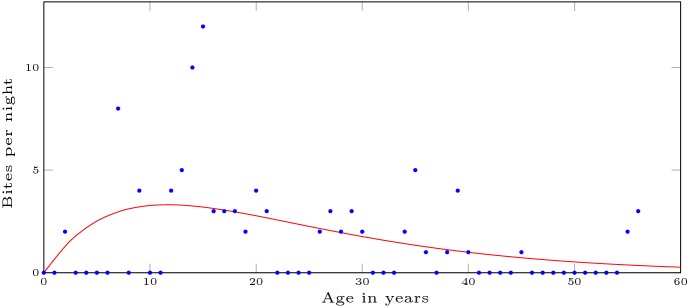
Biting rate data (Massad 2015) where bites per night are recorded for individuals aged 0–56 years (dots) together with the fitted age-dependent biting rate of the form $q(a) = k_1ae^{-k_2a}$ (line)

We assume that the rate at which mosquitoes bite humans of a certain age does not depend on the specific serotype and based on fitting mosquito biting data from Brazil to a function $q(a) = k_1ae^{-k_2a}$, as shown in Fig. 1, is given by

11 $$\begin{aligned} q(a) = 282.7ae^{-0.08593a} \end{aligned}$$

where *a* is given in years. With this biting rate, the survival probability $\pi (a)$ as given in Eq. (2), and by setting $C(a)\equiv 1$ as an approximation the double integrals in both expressions of $R_0$ can be solved analytically. For Eqs. (9) and (10) one obtains

12 $$\begin{aligned} R_0 \approx \frac{mbce^{-\mu _M\tau }}{\mu _ML}\frac{k_1^2\left( 4k_2^3\omega _0 + \left( k_2-\gamma _H\right) ^2\left[ \left( k_2+\gamma _H\right) \omega _1+k_2\omega _2\right] \right) }{4k_2^3\left( k_2+\gamma _H\right) ^2\left( k_2-\gamma _H\right) ^2}, \end{aligned}$$

and

13 $$\begin{aligned} R_0 \approx e^{\lambda \tau } \frac{\left( \mu _M+\lambda \right) \left( k_2+\gamma _H+\lambda \right) ^2\left( k_2-\gamma _H-\lambda \right) ^2\left( 4k_2^3\omega _0 + \left( k_2-\gamma _H\right) ^2\left[ \left( k_2+\gamma _H\right) \omega _1+k_2\omega _2\right] \right) }{\mu _M\left( k_2+\gamma _H\right) ^2\left( k_2-\gamma _H\right) ^2\left( 4k_2^3\omega _3 - \left( k_2+\gamma _H+\lambda \right) ^2\left[ \left( k_2-\gamma _H-\lambda \right) \omega _1+k_2\omega _2\right] \right) } , \end{aligned}$$

respectively, where

14 $$\begin{aligned} \omega _0&= \left( \left( k_2L+1\right) ^2-\gamma _H^2L^2\right) e^{-2k_2L}-\left( \left( k_2+\gamma _H\right) L+1\right) e^{-\left( k_2+\gamma _H\right) L},\nonumber \\ \omega _1&= 1-\left( \left( k_2L+1\right) 2k_2L+1\right) e^{-2k_2L},\nonumber \\ \omega _2&= 1-\left( 2k_2L+1\right) e^{-2k_2L}, \text { and}\nonumber \\ \omega _3&= 1-\left( \left( k_2+\gamma _H+\lambda \right) L+1\right) e^{-\left( k_2+\gamma _H+\lambda \right) L}. \end{aligned}$$

Note that since $\gamma _HL$ is the average human lifetime divided by the average infectious period and therefore very large, $\gamma _H \gg k_2,\lambda$ terms including $e^{-\gamma _HL}$ can be neglected so that $\omega _0 \approx ((k_2L+1)^2-\gamma _H^2L^2)e^{-2k_2L}$, and $\omega _3 \approx 1$.

### Serotype-Specific Basic Reproduction Numbers

The basic reproduction number for each serotype DENv1–4 can now be approximated using Eq. (13). Values for the model parameters $\gamma _H = 0.14~\hbox {day}^{-1}$, $\mu _M = 0.025~\hbox {day}^{-1}$, and $L=73.8$ years can be found in the literature (Massad et al. 2010; United States Central Intelligence Agency 2016) and are assumed non-specific for the different serotypes. $k_2 = 0.08593~\hbox {year}^{-1}$ was found from data as mentioned before and is also assumed equal for all four serotypes. The initial growth rate $\lambda$ on the other hand is serotype specific and can be found for each serotype at the beginning of an epidemic by fitting the number of new cases of that type to an exponential curve (Massad et al. 2010; Favier et al. 2006).

The Brazilian Ministry of Health (SINAN) recorded the number of dengue cases by serotype in the years from 2000 to 2014 for the regions North, Northeast, South, Southeast, and Centre West. To obtain a serotype-specific $\lambda$ and hence $R_0$ we determined the first 12 weeks of each epidemic caused by a given serotype for the whole of Brazil. The corresponding data for each of the regions were then used to find upper and lower bounds for $\lambda$ and $R_0$. During the surveyed period DENv1–3 each caused four major nationwide outbreaks, while DENv4 only caused two. The serotype-specific reproduction numbers are taken to be the mean of the basic reproduction numbers of all outbreaks caused by that type and are given alongside their upper and lower bounds in Table 2 where the upper and lower bounds are the highest and lowest $R_0$ values obtained for that serotype in any region. While these results seem to be in agreement with previous serotype-specific estimates (Coudeville and Garnett 2012; Reiner et al. 2014) it is important to note that there is a significant dependence on which weeks are considered to correspond to the outbreak. This is due to the fact that the initial outbreak of an epidemic cannot be ascertained beyond doubt from the data due to slight differences in the starting point of epidemics caused by the different climatic conditions in each region.

**Table 2:** Serotype-specific basic reproduction numbers

Serotype	$R_0$	Lower bound	Upper bound
DENv1	4.7045	1.2230	6.1777
DENv2	2.9942	1.3745	8.5133
DENv3	4.2974	1.4341	13.4129
DENv4	4.1864	1.8291	4.8711

## Risk of Infection

Our goal is to apply the transmission model to find optimal vaccination ages. To do this we need to identify which consequences of an infection vaccination aim to minimise. This can be achieved by defining the lifetime expected risk of dengue, i.e. the total risk from infection during the lifetime of an individual by considering the expected risk from infection with any one serotype.

### Expected Risk from Infection with Serotype *i* at Age *a*

To define the expected risk we assume that the transmission dynamics have reached a steady state. The steady-state force of infection is then $\lambda (a) = \lim _{t \rightarrow \infty }\lambda _H(a,t)$, while the steady-state age distribution of unaffected humans is given by $U(a) = \lim _{t \rightarrow \infty }U_H(a,t)$. The probability of being unaffected at age *a* which is denoted by *u*(*a*) can therefore be obtained by taking the fraction of unaffected with respect to all humans of age *a*, i.e. $u(a) = U(a)/N(a)$, where $N(a) = \frac{N_H}{L}\pi (a)$ is the equilibrium density with respect to age of the human population. By considering the matching conditions the fraction of successfully vaccinated individuals *v*(*a*) can be obtained as

15 $$\begin{aligned} v(a)&= {\left\{ \begin{array}{ll} 0, &{} 0\le a\le A_1, \\ q_1u(A_1^-), &{} A_1< a\le A_2, \\ q_1u(A_1^-)+q_2u(A_2^-), &{} A_2< a\le A_3, \\ q_1u(A_1^-)+q_2u(A_2^-)+q_3u(A_3^-), &{} A_3< a< \infty , \end{array}\right. } \end{aligned}$$

where $q_i=V_iC(A_i)$ is the probability of becoming immune due to vaccination at age $A_i$ for $i=1,2,3$. Consequently $1-\left( u(a)+v(a)\right)$ is the probability of having been infected before reaching age *a*. We further obtain the fraction of unaffected who become infected on exposure to be *C*(*a*)*u*(*a*) and therefore the probability of infection at age *a* as $P(a)=\lambda (a)C(a)u(a)$.

If there is some risk *R*(*a*) assigned to being infected at age *a* that describes how undesirable an infection at that age is we can obtain the expected risk from an infection at age *a* as

16 $$\begin{aligned} E(a)= P(a)R(a) = \lambda (a)C(a)u(a)R(a). \end{aligned}$$

Note that there are several dengue serotypes so that an infection at age *a* might be a primary, secondary, tertiary, or quaternary infection depending on how many serotypes coexist in an endemic region and whether the infected individual was previously infected by or vaccinated against any of them. If the risk of each of these types of infection was the same the expected risk at age *a* could be described by Eq. (16). However, since there is substantial evidence that secondary infections cause more severe infections and some indication that the sequence of serotypes plays an important role (Halstead 2009; Fried et al. 2010; Gibbons et al. 2007) the expected risk at age *a* depends on how many and which serotypes an individual was previously infected with. Additionally it has been observed that the risk varies depending on the serostatus of a vaccine recipient (SAGE/World Health Organization 2016). We therefore need to find the probabilities of an infection with a given serotype being a primary, secondary, tertiary, or quaternary infection when previous exposure was caused by natural infection or vaccination. Note that vaccination is considered to be a silent infection. These probabilities are calculated as

17 $$\begin{aligned} P_{ijkl}(a)&= \lambda _i(a)C_i(a)u_i(a)(1-(u_j(a)+v_j(a)))(1-(u_k(a)\nonumber \\&\quad +v_k(a)))(1-(u_l(a)+v_l(a))),\nonumber \\ P_{ijk\bar{l}}(a)&= \lambda _i(a)C_i(a)u_i(a)(1-(u_j(a) +v_j(a)))(1-(u_k(a)+v_k(a)))u_l(a),\nonumber \\ P_{ij\bar{k}\bar{l}}(a)&= \lambda _i(a)C_i(a)u_i(a)(1-(u_j(a)+v_j(a)))u_k(a)u_l(a),\nonumber \\ P_{i\bar{j}\bar{k}\bar{l}}(a)&= \lambda _i(a)C_i(a)u_i(a)u_j(a)u_k(a)u_l(a),\nonumber \\ P_{ijkl_*}(a)&= \lambda _i(a)C_i(a)u_i(a)(1-(u_j(a)+v_j(a)))(1-(u_k(a)+v_k(a)))v_l(a),\nonumber \\ P_{ijk_*l_*}(a)&= \lambda _i(a)C_i(a)u_i(a)(1-(u_j(a)+v_j(a)))v_k(a)v_l(a),\nonumber \\ P_{ij_*k_*l_*}(a)&= \lambda _i(a)C_i(a)u_i(a)v_j(a)v_k(a)v_l(a),\nonumber \\ P_{ijk_*\bar{l}}(a)&= \lambda _i(a)C_i(a)u_i(a)(1-(u_j(a)+v_j(a)))v_k(a)u_l(a),\nonumber \\ P_{ij_*k_*\bar{l}}(a)&= \lambda _i(a)C_i(a)u_i(a)v_j(a)v_k(a)u_l(a),\nonumber \\ P_{ij_*\bar{k}\bar{l}}(a)&= \lambda _i(a)C_i(a)u_i(a)v_j(a)u_k(a)u_l(a), \end{aligned}$$

where *j*, $j_*$, and $\bar{j}$ indicate, respectively, a previous natural infection with serotype *j*, successful vaccination against serotype *j* before age *a*, and no previous infection with or vaccination against serotype *j*, that is, $P_{ijk_*\bar{l}}(a)$ denotes the probability of an infection with serotype *i* at age *a* after a previous infection with serotype *j* and successful vaccination against serotype *k* but no exposure to serotype *l*. By denoting the corresponding risk functions in a similar manner, e.g. $R_{ijk_*\bar{l}}(a)$, the expected risk from an infection with serotype *i* at age *a* can then be calculated as

18 $$\begin{aligned}&E_i(a) = P_{i\bar{j}\bar{k}\bar{l}}(a)R_{i\bar{j}\bar{k}\bar{l}}(a) + P_{ij\bar{k}\bar{l}}(a)R_{ij\bar{k}\bar{l}}(a) + P_{i\bar{j}k\bar{l}}(a)R_{i\bar{j}k\bar{l}}(a) + P_{i\bar{j}\bar{k}l}(a)R_{i\bar{j}\bar{k}l}(a)\nonumber \\&\quad + P_{ij_*\bar{k}\bar{l}}(a)R_{ij_*\bar{k}\bar{l}}(a) + P_{i\bar{j}k_*\bar{l}}(a)R_{i\bar{j}k_*\bar{l}}(a) + P_{i\bar{j}\bar{k}l_*}(a)R_{i\bar{j}\bar{k}l_*}(a) + P_{ijk\bar{l}}(a)R_{ijk\bar{l}}(a)\nonumber \\&\quad + P_{ij\bar{k}l}(a)R_{ij\bar{k}l}(a) + P_{i\bar{j}kl}(a)R_{i\bar{j}kl}(a) + P_{ij_*k_*\bar{l}}(a)R_{ij_*k_*\bar{l}}(a) + P_{ij_*\bar{k}l_*}(a)R_{ij_*\bar{k}l_*}(a)\nonumber \\&\quad + P_{i\bar{j}k_*l_*}(a)R_{i\bar{j}k_*l_*}(a) + P_{ijk_*\bar{l}}(a)R_{ijk_*\bar{l}}(a) + P_{ij\bar{k}l_*}(a)R_{ij\bar{k}l_*}(a) + P_{ij_*k\bar{l}}(a)R_{ij_*k\bar{l}}(a)\nonumber \\&\quad + P_{ij_*\bar{k}l}(a)R_{ij_*\bar{k}l}(a) + P_{i\bar{j}kl_*}(a)R_{i\bar{j}kl_*}(a)+ P_{i\bar{j}k_*l}(a)R_{i\bar{j}k_*l}(a) + P_{ijkl}(a)R_{ijkl}(a)\nonumber \\&\quad + P_{ijkl_*}(a)R_{ijkl_*}(a) + P_{ijk_*l}(a)R_{ijk_*l}(a) + P_{ij_*kl}(a)R_{ij_*kl}(a) + P_{ijk_*l_*}(a)R_{ijk_*l_*}(a)\nonumber \\&\quad + P_{ij_*kl_*}(a)R_{ij_*kl_*}(a) + P_{ij_*k_*l}(a)R_{ij_*k_*l}(a) + P_{ij_*k_*l_*}(a)R_{ij_*k_*l_*}(a). \end{aligned}$$

The risks associated with the probabilities in Eq. (17) depend on several assumptions and can be defined based on any negative effect an infection may have. The risk functions are a measure of the undesirability of having dengue at a certain age, and many definitions are possible. Considering that only a fraction of dengue cases lead to severe symptoms while the majority show only mild symptoms or are completely asymptomatic (Gubler 2011; Bhatt et al. 2013) the risk functions should be defined in such a way that the burden of dengue is adequately described. We will consider the risk of being admitted to hospital due to an infection. In this case the pre-vaccine risk of hospitalisation can be derived from data provided by SINAN that was reviewed by Burattini et al. (2016) to evaluate age differences in hospital admissions due to dengue. The data show that the highest risk is associated with young ages, with a peak at approximately 5.5 years. Adults have a relatively low risk of requiring hospitalisation, but a significant increase in risk at ages above 70 years occurs. The risk function *R*(*a*) describing the undesirability of acquiring dengue at age *a* in years based on the need for hospital treatment can therefore be obtained by fitting a piecewise defined function to the data. Based on the data the function at young ages is assumed to be of the form $k_1ae^{-k_2a}$ and at older ages it is an exponential function of the type $l_1e^{l_2a}$. For ages above the highest recorded age the risk is taken to be constant. The pre-vaccine risk of hospitalisation is then given by:

19 $$\begin{aligned} R(a)&= {\left\{ \begin{array}{ll} 0.09153ae^{-0.1820a}, &{} 0 \le a< 21.3333, \\ 0.02428e^{0.02362a}, &{} 21.3333 \le a< 100, \\ 0.02428e^{0.02362\cdot 100},&{} 100 \le a < \infty . \end{array}\right. } \end{aligned}$$

If we assume that the risk of a secondary, tertiary, and quaternary infection does not depend on whether the previous infections were natural infections or silent vaccine-induced infections, then the associated risks can easily be defined depending on whether ADE and permanent cross-immunity after two heterologous infections are considered, that is, if there is ADE, we assume primary infections to be risk-free, i.e. $R_{i\bar{j}\bar{k}\bar{l}}(a) = 0$ and all remaining infections to have the same risk which is approximated by *R*(*a*). Similarly if there is no ADE and a secondary infection with a heterologous serotype confers permanent cross-immunity, we assume primary and secondary infections to have the same risk as before the introduction of the vaccine, and tertiary and quaternary infections to be risk-free, i.e. $R_{ijkl}(a) = R_{ijk\bar{l}}(a) = R_{ijkl_*}(a) = R_{ijk_*l_*}(a) = R_{ij_*k_*l_*}(a) = R_{ijk_*\bar{l}}(a) = R_{ij_*k_*\bar{l}}(a) = 0$. However, results from the long-term follow-up of Dengvaxia trials show an increased risk of hospitalisation in seronegative vaccine recipients (SAGE/World Health Organization 2016; Martínez-Vega et al. 2017; Aguiar and Stollenwerk 2017). Based on these results the associated risk functions in the case of serostatus-dependent risk can be derived as outlined in Supplementary Appendix B.

### Lifetime Expected Risk *E*

Using the definition of the expected risk from an infection given by Eq. (18) we can now define the lifetime expected risk *E* as the integral over the sum of the expected risks of the different serotypes multiplied by the survival probability $\pi (a)$ over all ages, i.e. we define

20 $$\begin{aligned} E = \int _0^{\infty }\left( \sum _{i=1}^{4}E_i(a)\right) \pi (a)\hbox {d}a. \end{aligned}$$

The incorporation of the survival probability is crucial since dengue does indeed affect people of all ages and the consequences are very grave if infections occur at old ages (Halstead 1980). This increased risk is often disregarded for infections that are typically observed during childhood which leads to an underestimation of the lifetime expected risk. On the other hand if the higher risk associated with an infection at old ages is taken account of, the survival probability is neglected the risk is overestimated. It is important to note that the inclusion of the survival probability in the lifetime expected risk significantly reduces the risk in the case of a step death function, i.e. due to the cut-off at $L=73.8$ years the high risk above this age does not factor in.

## Steady-State Dynamics

In the previous section we introduced the lifetime expected risk of an infection with dengue. The definition of this risk is based on the assumption that a steady-state age distribution has been reached. We therefore need to find both the steady-state age distributions and the steady-state force of infection for the human population. At the steady state, the age distributions of the humans and the number of mosquitoes in each compartment are constant in time. The age distributions of unaffected, infected, and recovered humans are denoted by $U(a) = \lim _{t \rightarrow \infty }U_H(a,t)$, $I(a) = \lim _{t \rightarrow \infty }I_H(a,t)$, and $R(a) = \lim _{t \rightarrow \infty }R_H(a,t)$, respectively. The steady-state force of infection is denoted by $\lambda (a) = \lim _{t \rightarrow \infty }\lambda _H(a,t)$.

### Steady-State Age Distribution

Consider the age distributions of the fractions of unaffected and infected humans at the steady state with respect to the age distribution of all humans, i.e. $u(a) = U(a)/N(a)$ and $i(a) = I(a)/N(a)$. At the steady state, with vaccination as previously described, these fractions satisfy the ordinary differential equations

21 $$\begin{aligned} \frac{\hbox {d}u}{\hbox {d}a}&=-\lambda (a)C(a)u(a),\nonumber \\ \frac{\hbox {d}i}{\hbox {d}a}&=\lambda (a)C(a)u(a)-\gamma _Hi(a), \end{aligned}$$

with the initial conditions $u(0)=1$ and $i(0)=0$, and the matching condition

$$\begin{aligned} \lim _{a \rightarrow A_i^+} u(a) = (1-V_iC(A_i))\lim _{a \rightarrow A_i^-} u(a) \end{aligned}$$

for each of the three vaccination ages.

This system can easily be solved to obtain

22 $$\begin{aligned} u(a) = {\left\{ \begin{array}{ll} e^{-\int _0^a\lambda (s)C(s)\hbox {d}s}, &{} 0\le a< A_1, \\ (1-V_1C(A_1))e^{-\int _0^a\lambda (s)C(s)\hbox {d}s}, &{} A_1\le a< A_2, \\ (1-V_1C(A_1))(1-V_2C(A_2))e^{-\int _0^a\lambda (s)C(s)\hbox {d}s}, &{} A_2\le a< A_3, \\ (1-V_1C(A_1))(1-V_2C(A_2))(1-V_3C(A_3))e^{-\int _0^a\lambda (s)C(s)\hbox {d}s}, &{} A_3\le a< \infty , \end{array}\right. } \end{aligned}$$

and

23 $$\begin{aligned} i(a)=e^{-\gamma _Ha}\int _0^a\lambda (s)C(s)u(s)e^{\gamma _Hs}\hbox {d}s. \end{aligned}$$

Hence, the steady-state age distributions are $U(a)=N(a)u(a)$ and $I(a)=N(a)i(a)$.

### Steady-State Force of Infection

The force of infection for the human population is given by Eq. (4). Considering that at the steady state the number of infectious mosquitoes is given by $I_M$ this yields the steady-state force of infection

24 $$\begin{aligned} \lambda (a) = \lim _{t \rightarrow \infty }\lambda _H(a,t) = q(a)\frac{b}{N_H}I_M. \end{aligned}$$

From the mosquito equations where the time derivatives are set to zero and by noting $S_M = N_M-(L_M+I_M)$ we have $\lambda _MS_M = \mu _M(L_M+I_M)$ and $\mu _MI_M = e^{-\mu _M\tau }\lambda _M S_M$ where $\lambda _M$ is the steady-state force of infection for the mosquito population. The number of infectious mosquitoes at the steady state is therefore given by $I_M = e^{-\mu _M\tau }N_M\frac{\lambda _M}{\mu _M+\lambda _M}$. Using Eq. (5) with the steady-state age distribution $I_H(a)=\frac{N_H}{L}i(a)\pi (a)$ for the infected humans and recalling $m=N_M/N_H$ the steady-state force of infection for humans is

25 $$\begin{aligned} \lambda (a) = q(a)mbe^{-\mu _M\tau }\dfrac{\dfrac{c}{L}\int _0^{\infty }q(a)i(a)\pi (a)\hbox {d}a}{\mu _M+\dfrac{c}{L}\int _0^{\infty }q(a)i(a)\pi (a)\hbox {d}a}. \end{aligned}$$

We expect the steady-state force of infection to be different for each of the four dengue serotypes. However, as mentioned in Sect. 3 the exact serotype-specific parameter values are not known. The steady-state force of infection can in fact be written in terms of the basic reproduction number as given by Eq. (12). It is then given by

26 $$\begin{aligned} \lambda (a) = q(a)\dfrac{\dfrac{4k_2^3\left( k_2+\gamma _H\right) ^2\left( k_2-\gamma _H\right) ^2}{4k_2^3\omega _o+\left( k_2-\gamma _H\right) ^2\left( \left( k_2+\gamma _H\right) \omega _1+k_2\omega _2\right) }R_0\int _0^{\infty }q(a)i(a)\pi (a)\hbox {d}a}{1+\dfrac{c}{\mu _ML}\int _0^{\infty }q(a)i(a)\pi (a)\hbox {d}a} \end{aligned}$$

where $\omega _0$, $\omega _1$, and $\omega _2$ are given in Eq. (14). By solving this expression using the serotype-specific basic reproduction numbers we found in Sect. 3.1 for a given vaccination strategy one can compute the lifetime expected risk given by Eq. (20).

## Results

We now want to find the optimal ages for vaccination against dengue with Dengvaxia for any combination of serotypes in an endemic region by numerically evaluating the lifetime expected risk derived in Sect. 4. This can be done by finding the serotype-specific forces of infection as given by Eq. (26) where it is important to note that since we assume independent transmission dynamics the combination of circulating serotypes does not influence the force of infection for each of the serotypes present.

Most of the parameters needed for the computation of the steady-state force of infections are already used in Sect. 3.1 to find the serotype-specific basic reproduction numbers, and the same values are used for the computation of the lifetime expected risk. The parameters that are still needed are the transmission probability from human to mosquito *c* and the fractions $V_i$ vaccinated at the ages $A_i$. For the probability of transmission we take $c=1$ from the literature (Massad et al. 2010) for all serotypes. The vaccination ages $A_i$ are such that the initial dose can be given at any age, while the second and third dose is given according to the licence of Dengvaxia, i.e. $A_2 = A_1 + 6$ months and $A_3 = A_1 + 12$ months. We do not restrict the initial age $A_1$ to the age range of the licence to find the optimal vaccination age. However, at the end of this section we briefly compare the optimal age and lifetime expected risk to what can be achieved under the current restrictions of the licence in Brazil, i.e. if vaccination takes place between the ages of 9 and 45 years.

The vaccinated fractions $V_i$ are utilised to incorporate the vaccine efficacy of Dengvaxia for each serotype by setting $V_i = 1-\left( 1- eff \right) ^{\frac{1}{3}}$ where $eff$ is the vaccine efficacy. Two phase three trials (Capeding et al. 2014; Hadinegoro et al. 2015) have shown that the efficacy depends on the serotype, the age of the recipient, and whether the recipient had a prior dengue infection or not. A summary of the different vaccine efficacies is given in Table 3. However, there is some dispute about the age dependence of the vaccine with some researchers, arguing that the increase in efficacy for older recipients is mainly due to the serostatus of the recipient (Halstead and Aguiar 2016; Halstead 2016; Aguiar et al. 2016; Flasche et al. 2016). The data from the long-term follow-up of the trials further indicate an increased risk of hospitalisation in seronegative recipients (Martínez-Vega et al. 2017; Aguiar et al. 2016). In addition to the constant, serotype-specific vaccine efficacy we will therefore consider vaccine-induced hospitalisation risk, as well as the effect of an age-dependent, serotype-specific vaccine efficacy.

**Table 3:** Vaccine efficacies as presented by Hadinegoro et al. (2015) and SAGE/World Health Organization (2016)

Vaccine efficacy	Age independent (%)	Under 9 years (%)	9 years or older (%)
*According to serotype*			
DENv1	54.7	46.6	58.4
DENv2	43.0	33.6	47.1
DENv3	71.6	62.1	73.6
DENv4	76.9	51.7	83.2
*According to serostatus*			
Seropositive	78.2	70.1	81.9
Seronegative	38.1	14.4	52.5

As mentioned in introduction, there is significant evidence that mainly secondary, heterologous infections are responsible for SD while primary infections are often asymptomatic. It is also believed that a secondary infection with a heterologous serotype confers permanent cross-immunity to all serotypes or at least that tertiary and quaternary infections are asymptomatic (Gibbons et al. 2007; Anderson et al. 2013). ADE and cross-immunity after two heterologous infections therefore impact the lifetime expected risk in addition to the vaccine efficacy and whether the risk is serostatus dependent. We therefore present results comparing the effect of assuming risky and risk-free primary infections, as well as symptomatic and asymptomatic third and fourth infections after two heterologous infections. Note that the tetravalence of the vaccine leads to the possibility of secondary, tertiary, and quaternary infections even if not all four serotypes coexist since the successful vaccination against any serotype can be considered a silent infection.

**Table 4:** Optimal vaccination age $A_1$ in months with the corresponding minimal lifetime expected risk *E* of hospitalisation due to dengue for unchanged risk in seronegative recipients and relative risks according to serostatus

3rd and 4th infections	Hospitalisation with serostatus-independent risk				Hospitalisation with serostatus-dependent risk			
	1st infection risky		1st infection risk-free		1st infection risky		1st infection risk-free	
	$A_1$	*E* ($\times 10^{-2}$)	$A_1$	*E* ($\times 10^{-2}$)	$A_1$	*E* ($\times 10^{-2}$)	$A_1$	*E* ($\times 10^{-2}$)
*Symptomatic*								
DENv1	14	7.12	–	0.00	–	16.84	–	0.00
DENv2	9	8.51	–	0.00	–	24.54	–	0.00
DENv3	14	4.13	–	0.00	258	17.25	–	0.00
DENv4	23	3.99	–	0.00	206	17.20	–	0.00
DENv12	11	15.64	76	11.57	126	40.81	147	33.26
DENv13	14	11.25	58	9.02	108	33.81	119	25.11
DENv14	18	11.20	63	8.33	111	33.77	120	24.69
DENv23	14	12.69	70	9.91	133	41.44	149	33.26
DENv24	17	12.66	70	9.24	135	41.66	149	33.10
DENv34	17	8.20	48	6.39	116	34.08	123	24.43
DENv123	14	19.80	48	17.33	94	56.53	106	54.75
DENv124	17	19.80	48	16.60	95	56.97	108	54.46
DENv134	17	15.34	42	13.00	94	51.20	102	45.80
DENv234	17	16.81	48	14.29	101	58.89	111	54.89
DENv1234	17	23.94	38	21.23	77	77.20	91	78.69
*Asymptomatic*								
DENv1	13	2.66	–	0.00	–	5,703	–	0.00
DENv2	14	2.70	–	0.00	–	37,169	–	0.00
DENv3	14	1.95	–	0.00	–	8,780	–	0.00
DENv4	17	1.92	–	0.00	–	9,296	–	0.00
DENv12	11	3.78	28	2.77	298	25,204	298	25,203
DENv13	12	3.66	28	2.67	289	10,679	289	10,678
DENv14	13	3.64	28	2.64	293	12,223	293	12,223
DENv23	12	3.60	28	2.63	307	39,766	307	39,765
DENv24	14	3.58	35	2.59	311	48,007	311	48,007
DENv34	14	3.37	28	2.43	307	19,181	307	19,181
DENv123	11	4.04	21	3.02	248	45.59	248	45.56
DENv124	11	4.03	24	3.01	254	50.91	254	50.88
DENv134	11	3.99	24	2.97	238	19.81	238	19.79
DENv234	11	3.95	24	2.94	265	75.17	265	75.14
DENv1234	10	4.19	21	3.16	156	1.69	156	1.68

Results both for risky and for risk-free primary infections are presented. In the bottom half the effect of permanent cross-immunity is considered by assuming asymptomatic third and fourth infections. The vaccine efficacy in all cases is assumed constant and serotype specific as given in Table 3.—corresponds to ages that lie outwith the age range of humans, i.e. vaccination in these cases is not recommended

### Constant Efficacy

Table 4 shows the obtained optimal vaccination age (in other words the optimal age of the first vaccination) and the corresponding minimal lifetime expected risk of a constant vaccine efficacy for all possible combinations of serotypes coexisting when vaccination aims at reducing the risk of hospitalisation without serostatus-dependent risk and of hospitalisation with serostatus-dependent risk. The optimal vaccination ages are very low if the vaccine is not assumed to increase the risk in seronegative recipients, independent of the number of serotypes, and any other assumptions. The only exception to this is if primary infections are assumed risk-free when only one serotype exists; in this case vaccination is not recommended. In the case of serostatus-dependent risk vaccination ages vary much more and depend significantly on whether third and fourth infections are asymptomatic or not. If primary infections are considered to be risk-free, vaccination is again not recommended for only one serotype. In addition vaccination is also not recommended for some scenarios with a single serotype in existence and risky primary infection. The assumption of ADE leads to a slight increase in optimal vaccination age for symptomatic third and fourth infections, but has no effect on the optimal vaccination age in the case of permanent cross-immunity.

**Fig. 2:**
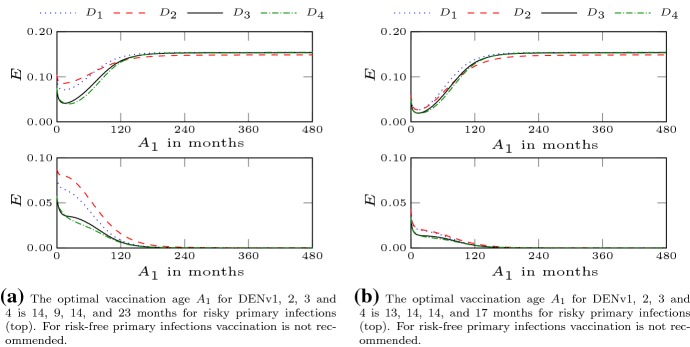
Lifetime expected risk *E* in an endemic area with a single serotype as a function of age $A_1$ in months at which the first of three doses of vaccine is administered for constant efficacy as given in Table 3 where third and fourth infections are assumed **a** symptomatic, and **b** asymptomatic. The graphs at the top show results for risky primary infections, while those at the bottom show results for risk-free primary infections. The risk associated with an infection is based on hospitalisation as given by Eq. (19)

**Fig. 3:**
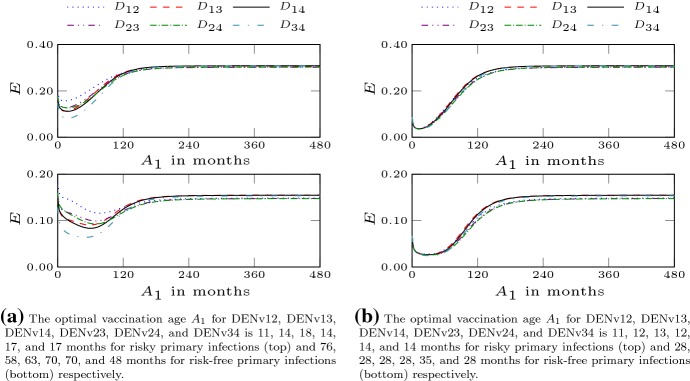
Lifetime expected risk *E* in an endemic area with two coexisting serotypes as a function of age $A_1$ in months at which the first of three doses of vaccine is administered for constant efficacy as given in Table 3 where third and fourth infections are assumed **a** symptomatic and **b** asymptomatic. The graphs at the top show results for risky primary infections, while those at the bottom show results for risk-free primary infections. The risk associated with an infection is based on hospitalisation as given by Eq. (19)

**Fig. 4:**
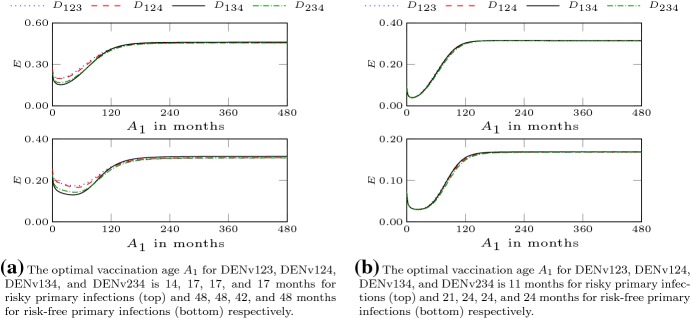
Lifetime expected risk *E* in an endemic area with three coexisting serotypes as a function of age $A_1$ in months at which the first of three doses of vaccine is administered **a** for symptomatic and **b** for asymptomatic tertiary infections. The graphs at the top show results for risky primary infections, while those at the bottom show results for risk-free primary infections. The risk associated with an infection is based on hospitalisation as given by Eq. (19)

**Fig. 5:**
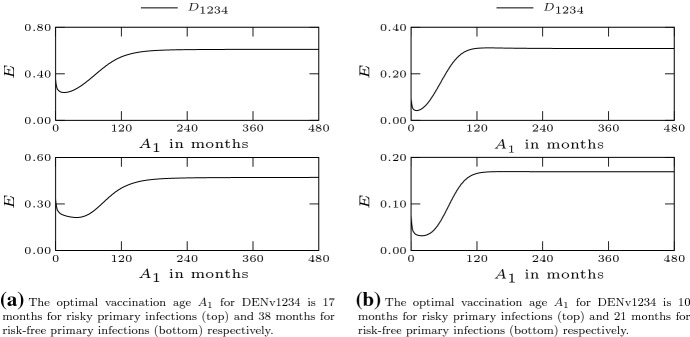
Lifetime expected risk *E* in an endemic area with all serotypes co-circulating as a function of age $A_1$ in months at which the first of three doses of vaccine is administered **a** for symptomatic and **b** for asymptomatic tertiary and quaternary infections. The graphs at the top show results for risky primary infections, while those at the bottom show results for risk-free primary infections. The risk associated with an infection is based on hospitalisation as given by Eq. (19)

We will begin by discussing in detail the results for constant efficacy when the risk does not depend on the serostatus of the recipient. For the different combinations of one, two, three, and four co-circulating serotypes the lifetime expected risks are shown in Figs. 2, 3, 4 and 5, respectively. For each combination of serotypes the figures present four different graphs, subfigure a shows results for symptomatic post-secondary infections, while subfigure b shows those for asymptomatic post-secondary infections. The top graph in both subfigures a and b considers risky primary infections, and the bottom graph risk-free primary infections. In each case the lifetime expected risk *E* is plotted against the vaccination age $A_1$ in months at which the first dose is administered. Since the goal is to minimise this risk the optimal vaccination age is the age $A_1$ with the lowest lifetime expected risk *E*. Note that in Figs. 2, 3, 4 and 5 the results shown on the left of Table 4 are presented, i.e. the vaccine efficacy is constant and only depends on the serotype, while the hospitalisation risk only depends on the age at infection but not the serostatus.

While in most endemic areas there are several coexisting serotypes, considering the case of a single serotype allows us to draw conclusions about the different vaccine efficacies and basic reproduction numbers. We therefore start by considering an endemic area where only a single serotype is present. The corresponding results are presented in Fig. 2. Vaccination is assumed to cause a silent infection if it is successful, so that due to the tetravalence of the vaccine secondary, tertiary, and quaternary infections are possible even if only one serotype circulates. If all infections are assumed equally risky, as shown in the top graph in Fig. 2a, the minimal lifetime expected risk is found at very young ages between 9 and 22 months for any of the serotypes. It can further be seen that for young vaccination ages the lifetime expected risk due to DENv4 is the lowest, followed by DENv3, DENv1, and lastly DENv2. On the other hand if vaccination is initiated at approximately 11.5 years or later, DENv2 causes the lowest lifetime expected risk. These observations can be explained by considering the serotype-specific basic reproduction numbers and vaccine efficacies as given in Tables 2 and 3. At young ages an effective vaccine can prevent a considerable number of cases even if the basic reproduction number is high so that the high vaccine efficacies for DENv3 and 4 (71.6% and 76.9%, respectively) lead to a low lifetime expected risk. On the other hand the lower efficacies for DENv1 and 2 with 54.7% and 43.0%, respectively, imply that even if vaccination occurs early, not many infections are prevented. The lifetime expected risk is therefore still fairly high and in fact highest for DENv2 even though it has the lowest basic reproduction number. If vaccination is given only to individuals aged 11.5 years or older, many infections will have already occurred, particularly if the basic reproduction number is high as is the case for serotypes 1, 3, and 4, so that vaccination will not reduce the overall risk. For DENv2 the basic reproduction number is significantly lower than for the remaining serotypes so that some cases in later life can be prevented even with the lower vaccine efficacy thus resulting in the lifetime expected risk due to DENv2 being the lowest at these high vaccination ages.

However, if we consider the first infection to be risk-free, we can see that the lifetime expected risk decreases with vaccination age for all serotypes. DENv2 always poses the highest risk due to the low efficacy for this serotype. With a step death function it makes sense to consider vaccination only within the age range 0–*L* years, where $L = 73.8$ years is the maximum human lifetime. Therefore, vaccination is not recommended if only one serotype exists but immunity caused by natural infection or vaccination leads to ADE. This can also intuitively be concluded, since if there is only one serotype, but primary infections are risk-free, there is no need to vaccinate at all. Now if instead of assuming symptomatic third and fourth infections we assume secondary infections to confer permanent cross-immunity, the corresponding results are shown in Fig. 2b. While the overall conclusions are very similar to the symptomatic case the lifetime expected risk is lower in general since a natural infection that occurs after successful vaccination against two other serotypes now no longer contributes to the lifetime expected risk. The optimal vaccination age decreases slightly to between 13 and 17 months in the case of risky primary infections. In this case it is particularly noticeable that the effects of the differences in basic reproduction number and efficacy of the serotypes are less pronounced if there is permanent cross-immunity. This is due to the fact that independent of circulating serotype successful vaccination against two serotypes means the natural infection will not be risky. For risk-free primary infections vaccination is again not recommended.

We now consider an endemic area with two co-circulating serotypes. The results are presented in Fig. 3. We shall start by considering all infections to be equally risky, i.e. the results presented in the graph at the top of Fig. 3a. We can see that similarly to one serotype existing very low vaccination ages are obtained which lie between 11 and 18 months. The effect of the vaccine efficacy is also similar to the case of a single serotype since the low efficacy of DENv2 leads to a higher lifetime expected risk whenever this serotype is present at young vaccination ages, while the combination of DENv3 and 4 leads to the lowest lifetime expected risk due the high combined efficacy. These observations are to be expected after considering the single serotype scenario and also apply in the case of risk-free primary infections as can be seen in the bottom graph. Assuming risk-free primary infections, however, leads to a lower lifetime expected risk and an optimal vaccination age between 48 and 76 months, i.e. much higher than for risky primary infections. The decrease in lifetime expected risk is caused by primary infections not contributing to the risk. The increase in vaccination age is due to fewer potentially risky infections at young ages, so that it is better to vaccinate later when maternal antibodies have declined. Comparing the lifetime expected risk of risky and risk-free primary infections shows that for risk-free primary infections there is a slightly wider range in which near optimal vaccination is possible.

Next we shall assume instead that tertiary and quaternary infections are asymptomatic, i.e. risk-free, as shown in Fig. 3b. As was the case for a single serotype it can immediately be seen that different efficacies and basic reproduction numbers have less effect in this case. Also the effect of assuming risk-free primary infections as opposed to risky ones becomes more pronounced, with a much wider range in which vaccination is near optimal in the former case and an increase in the optimal vaccination age from between 11 and 14 months to between 28 and 35 months. It can therefore be noted that assuming risk-free primary infections in general increases the optimal vaccination age, while assuming asymptomatic, i.e. risk-free, tertiary, and quaternary, infections decreases the optimal age particularly if primary infections are considered risk-free. For risk-free primary infections the optimal age increases since the first infection does not need to be prevented; in fact, if vaccination is only successful against one serotype, it is better to wait. On the other hand if third and fourth infections are asymptomatic vaccinating after a secondary infection will be useless so that in this case the optimal ages decrease.

We now increase the number of serotypes in an endemic area to three. The results for this scenario are presented in Fig. 4. Again we start by assuming that all infections are equally risky, i.e. symptomatic; then, from the top of Fig. 4a we can see that the optimal vaccination ages are between 14 and 17 months which is similar to the cases of one or two coexisting serotypes. Again it can be seen that for low vaccination ages the combination of DENv1, 3, and 4 has the lowest lifetime expected risk since this combination has the highest combined efficacy, and the combination of DENv1, 2, and 3 leads to the highest lifetime expected risk since the combined efficacy is lowest. Therefore, the same observations are made as for one or two coexisting serotypes. This is also true with respect to risk-free primary infections and symptomatic third and fourth infections as shown at the bottom of Fig. 4a. Again the optimal vaccination ages increase to between 42 and 48 months, and there is a wider range in which near optimal vaccination is possible for the reasons previously discussed. For asymptomatic third and fourth infections as shown in Fig. 4b the vaccination ages are 11 months for risky primary infections and between 21 and 24 months for risk-free primary infections. Again the graphs clearly show that the differences in efficacy and basic reproduction number are less decisive for the lifetime expected risk than in the case of symptomatic tertiary and quaternary infections. Similar to the case of two coexisting serotypes it can also be seen that the age range in which near optimal vaccination is possible increases more significantly for asymptomatic third and fourth infections if risk-free primary infections are considered than in the case of symptomatic ones.

Finally consider an endemic area with all four dengue serotypes DENv1–4 coexisting. The results for this case are presented in Fig. 5. The optimal vaccination age obtained for symptomatic tertiary and quaternary infections is 17 months in the case of risky primary infections and 38 months for risk-free ones. For asymptomatic third and fourth infections the corresponding optimal vaccination ages are 10 and 21 months. The lifetime expected risk is lower for risk-free primary infections as was the case for less co-circulating serotypes. The assumption of risk-free primary infections leads to a higher optimal vaccination age than for risky primary infection with a wider range in which near optimal vaccination is possible, and assuming permanent cross-immunity after two heterologous infections results in a significant decrease in optimal vaccination age. The results for four serotypes are therefore as expected from considering less coexisting serotypes.

### Serostatus-Dependent Risk

One of the most challenging aspects of dengue vaccination is the potential for an increased risk caused by the vaccine itself. From the long-term follow-up of the Dengvaxia trials it has been shown that initially seronegative recipients may experience a higher risk of hospitalisation in breakthrough cases after vaccination (SAGE/World Health Organization 2016; Martínez-Vega et al. 2017; Aguiar et al. 2016). Based on the data from these trials we therefore consider serostatus-dependent hospitalisation risk by determining the risk functions which we defined in Sect. 4 as described in Supplementary Appendix B. The minimal lifetime expected risk and optimal vaccination age for any number and combination of serotypes and for the different assumptions relating to ADE and cross-immunity are presented on the right of Table 4. Note that the vaccine efficacy used in the matching conditions is still assumed constant and only depends on the serotype, while the hospitalisation risk now depends on both the age at infection and the serostatus just prior to infection. In the case of serostatus-dependent risk, vaccination of an individual who was initially seronegative is generally not desirable unless they are successfully vaccinated against all endemic serotypes. It is therefore not surprising that the optimal vaccination age increases, so as to vaccinate less seronegatives compared to the case of no serostatus-dependent risk. However, if two heterologous infections confer permanent cross-immunity, it can still be beneficial to vaccinate seronegatives if they are successfully vaccinated against at least two serotypes since subsequent natural infections will be asymptomatic. For risk-free primary infections but symptomatic third and fourth infections it is least desirable to vaccinate seronegatives because successful vaccination against less than four serotypes will increase their risk in every breakthrough natural infection.

**Fig. 6:**
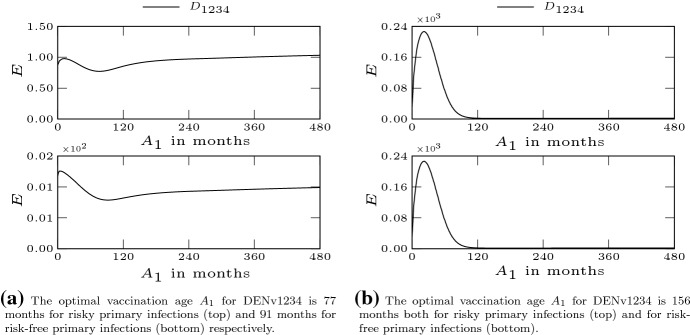
Lifetime expected risk *E* in an endemic area with all serotypes co-circulating as a function of age $A_1$ in months at which the first of three doses of vaccine is administered **a** for symptomatic and **b** for asymptomatic tertiary and quaternary infections. The graphs at the top show results for risky primary infections, while those at the bottom show results for risk-free primary infections. The risk associated with an infection is based on hospitalisation as given by Eq. (19). Additionally vaccination induces an increased risk in seronegative recipients according to Table B.1 (see Supplementary Appendix B)

We will briefly consider the consequences of a serostatus-dependent risk on the basis of all four serotypes coexisting as shown in Fig. 6.

By comparing Figs. 5 and 6 we can see that serostatus-dependent risk has a significant effect on the lifetime expected risk. Particularly at young ages, vaccination with a vaccine that induces an additional risk in seronegative recipients leads to a higher lifetime expected risk. Once the vaccination age increases to ages in which few individuals are seronegative the effect is much less pronounced. The optimal vaccination ages independent of the assumptions to ADE and permanent cross-immunity are therefore higher with a vaccine-induced risk. For symptomatic third and fourth infections it can further be seen that the optimal vaccination age increases slightly if primary infections are assumed risk-free. If post-secondary infections are asymptomatic, there is hardly any difference between risky and risk-free primary infections. In this case vaccinating at early ages increases the lifetime expected risk significantly independent of whether ADE is considered or not. Independent of whether primary infections are risky or not it is therefore ideal to vaccinate after the first natural infection occurred if post-secondary infections are asymptomatic. This has two reasons: Firstly, seropositive individuals are not exposed to an increased risk by vaccination so that it is better to wait for more individuals to have had a natural infection. Secondly, if a seropositive individual is successfully vaccinated against any serotype, they were not infected with permanent cross-immunity and will protect them against all future infections. From Fig. 6b it can be seen that the optimal vaccination age is 13 years for permanent cross-immunity independent of the assumption relating to ADE; 13 years is therefore the age at which most primary infections but few secondary infections will have occurred.

### Age-Dependent Vaccine Efficacy

So far only results for constant vaccine efficacies were presented. However, as mentioned before, Hadinegoro et al. (2015) found the vaccine efficacies to increase for recipients aged 9 years or older. This phenomenon is believed to be caused by the serostatus of the recipient rather than their age and the constant efficacy can therefore be considered to be more accurate. Additionally, if the vaccine does not induce a higher risk in seronegative recipients, the optimal ages in the case of age-dependent vaccine efficacy are in fact very similar to those presented in Table 4 for constant vaccine efficacy. We will therefore only briefly highlight the differences between constant and age-dependent vaccine efficacy results by considering a single serotype in existence and no serostatus-dependent risk.

**Fig. 7:**
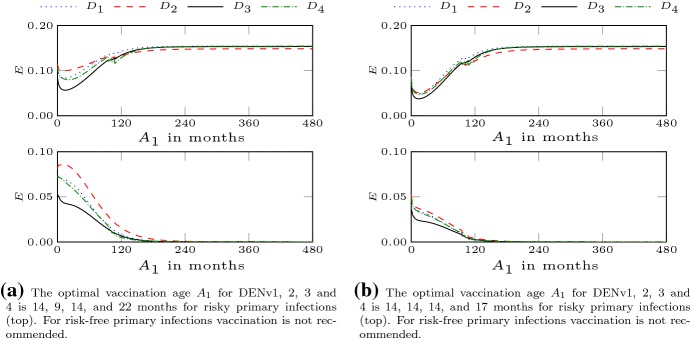
Lifetime expected risk *E* in an endemic area with a single serotype as a function of age $A_1$ in months at which the first of three doses of vaccine is administered for vaccine efficacy depending on the age groups $<9$ and $\ge 9$ years as shown in Table 3 where third and fourth infections are assumed **a** symptomatic and **b** asymptomatic. The graphs at the top show results for risky primary infections, while those at the bottom show results for risk-free primary infections. The risk associated with an infection is based on hospitalisation as given by Eq. (19)

If the vaccine efficacy for the serotypes is based on the age groups as given in Table 3 the resulting lifetime expected risk of a single serotype is shown in Fig. 7. For all serotypes the efficacy based on age groups is lower for children under the age of 9 years and higher for individuals aged 9 years or older in comparison with the constant efficacies. While the observations of the constant efficacy case also apply to the age-dependent case, the increase in efficacy for any of the three vaccination doses leads to a drop in lifetime expected risk which can be seen at ages $A_1 \in \{96, 102, 108\}$ months. The drops are more pronounced in the case of risky primary infections both for symptomatic and for asymptomatic third and fourth infections. Despite the highest efficacy being reached once all three doses are given above the age of 9 years, i.e. once $A_1 \ge 108$ months, the optimal vaccination ages remain very low for risky primary infections independent of the symptomaticity of tertiary and quaternary infections. In fact they differ by no more than 1 month from the constant efficacy case. The lower vaccine efficacy in under 9 years old is also responsible for the higher lifetime expected risk in this age-group in comparison with the constant efficacy results. For risk-free primary infections vaccination is again not recommended. Similar effects can be observed for more than one serotype in circulation. However, it is important to note that the increase in efficacy at 9 years has a significant impact on the optimal vaccination ages in the case of two serotypes coexisting when primary infections are risk-free and post-secondary infections are symptomatic. The reason for this is that the optimal vaccination ages for constant efficacy in this case are already fairly high as shown in Table 4; the increased efficacy at 9 years decreases the lifetime expected risk sufficiently to lead to a higher optimal vaccination age of exactly 9 years for most combinations of two serotypes.

### Licence Restrictions

The current licence of Dengvaxia only allows vaccination of individuals aged between 9 and 45 years in Brazil. However, when vaccination does not induce an additional risk the optimal vaccination age for the risk of hospitalisation lies between 9 and 76 months for almost any scenario as shown in Table 4. The only exception is one circulating serotype and risk-free primary infections in which case the optimal vaccination policy was not to vaccinate. The optimal vaccination ages are therefore significantly below the permitted age range if there is no serostatus-dependent risk. In the case of serostatus-dependent risk only very few optimal vaccination ages are below 9 years. However, the optimal vaccination ages in this case are much closer to the permitted age range than when the vaccine is not assumed to induce any additional risk. If the age at which the first dose of the vaccine can be administered is restricted such that all doses are given to individuals aged 9–45 years, the optimal vaccination age is 108 months in all cases in which vaccination is recommended independent of the assumption regarding the serostatus-dependent risk. However, restricting the vaccination age as required by the licence leads to a significant increase in lifetime expected risk compared to what could be achieved if vaccination was possible at younger ages. For cases in which vaccination is recommended the increase in the lifetime expected risk compared to its optimum as given in Table 4 lies between approximately 7% and 630% without a vaccine-induced risk and below 7% when such a risk is considered. The percentage increase is higher the further from the optimal age vaccination takes place under the age restriction.

## Discussion

In this paper we found the optimal vaccination age for dengue vaccination in Brazil according to a routine vaccination calendar in endemic regions with any number and combination of dengue serotypes. We assumed independent transmission dynamics for the different serotypes and derived both an exact and an approximate expression for the basic reproduction number of our model. From data we then found serotype-specific basic reproduction numbers, an age-dependent mosquito biting rate, and an age-dependent risk function describing the undesirability of getting an infection in terms of the risk of hospitalisation. The vaccine efficacy in the transmission model was assumed serotype specific, results corresponding to constant efficacy were presented in detail while those corresponding to age-dependent efficacy were presented only for the risk of hospitalisation in an area with one serotype and briefly addressed otherwise. In addition we discussed the effect of an increased risk in seronegative recipients. Risk-free primary infections corresponding to ADE and permanent cross-protection after two heterologous infections were considered. For optimal vaccination ages that did not match the current age range of 9–45 years for which Dengvaxia can be used in Brazil we further determined the optimal vaccination age under these constraints.

The optimal vaccination ages which we obtained for constant efficacy in the case of hospitalisation and hospitalisation with a serostatus-dependent risk are given in Table 4 alongside the minimal lifetime expected risk. We found that optimal vaccination ages if there is no serostatus-dependent risk are very low. For risky primary infections the optimal age is between 9 and 23 months, while for risk-free primary infections it is between 21 and 76 months when vaccination is recommended. The increase in optimal vaccination age if primary infections are considered risk-free can be explained by considering that in this case vaccination is beneficial only after a primary but before a secondary infection. On the one hand this is because at younger ages it may be given to children who are still protected by maternal antibodies and will thus not be effective. On the other hand if vaccination is successful only against one serotype, the first natural infection will correspond to a secondary infection and thus result in a higher risk. This is also the reason why vaccination is not recommended if only a single serotype is in circulation and primary infections are risk-free, i.e. dengue infections are subject to ADE. The assumption of asymptomatic tertiary and quaternary infections, i.e. secondary infections effectively conferring permanent cross-immunity, resulted in lower optimal vaccination ages than those found for corresponding results with symptomatic infections. This change is larger when the assumption is that primary infections are risk-free since in this case vaccination is only beneficial if it prevents secondary infections.

Dengvaxia was first licensed in December 2015. Since the introduction of Dengvaxia in endemic countries concerns about its application in seronegative recipients have been raised due to an increase in SD cases in those recipients, and the need for pre-vaccination screening has been discussed (Halstead and Aguiar 2016; Halstead 2016; Aguiar et al. 2016; Flasche et al. 2016). Considering the increased hospitalisation risk in seronegative recipients that was observed in the long-term follow-up of Dengvaxia trials (SAGE/World Health Organization 2016; Martínez-Vega et al. 2017; Aguiar et al. 2016), we considered serostatus-dependent risk by calculating relative risks according to serostatus as described in Supplementary Appendix B. The optimal vaccination ages and lifetime expected risks are presented in Table 4. With an imperfect vaccine and an increased risk in breakthrough infections of initially seronegative recipients it can be counterproductive to vaccinate seronegative recipients. It is therefore not surprising that in the case of serostatus-dependent risk the optimal vaccination ages were found to be much higher in every case. This is due to the fact that ideally vaccination takes place after most primary infections occurred to prevent exposing vaccinated individuals to any additional risk. More circulating serotypes generally reduce the average age of a primary infection; therefore, the optimal vaccination age decreases slightly as the number of serotypes increases. Considering that vaccination is already carried out at an age at which few individuals are seropositive it is not surprising that the assumption of ADE has little effect on the optimal vaccination age. Most of the obtained optimal vaccination ages in the case of a serostatus-dependent risk are in the permitted age range of Dengvaxia. Again, for risk-free primary infections and only a single serotype in existence it is best not to vaccinate. In addition some combinations of a single serotype in existence with risky primary infections are also better not targeted by vaccination.

The vaccine efficacy of Dengvaxia was found to be age dependent in several Phase Three trials (Capeding et al. 2014; Hadinegoro et al. 2015), and we therefore repeated all simulations using the age-dependent efficacies and presented the results for cases with one serotype and no serostatus-dependent risk. For the remaining cases the results were briefly discussed. The optimal vaccination ages are found to be very similar to those presented in Table 4 with the exceptions of most combinations of two coexisting serotypes if primary infections are risk-free and tertiary and quaternary infections symptomatic. In these cases the optimal vaccination age was found to be 108 months, i.e. 9 years. These are in fact the only scenarios for which we found the optimal vaccination age to be in the age range for which Dengvaxia is licensed in Brazil if there is no serostatus-dependent risk.

If the vaccination age is restricted to between the ages of 9 and 45 years, the optimal vaccination age is 108 months for all cases in which vaccination is recommended, irrespective of all other assumptions. This vaccination age is the earliest possible age at which vaccination is permitted under the licence restrictions and thus as soon as possible after the actual optimal vaccination age. The lifetime expected risk at this age is significantly higher than at the optimum. In general the later vaccination can take place after the optimal vaccination age the higher the percentage increase from the minimal lifetime expected risk is.

The most likely scenario in the majority of endemic areas in Brazil, and in fact in the world, is that all four dengue serotypes coexist. Based on the evaluation of nearly 7 million cases leading to hospitalisation in Brazil (Burattini et al. 2016) our conclusion is that primary infections are in fact risky. This does not mean that ADE does not play an important role in the risk of dengue, but there is a trade-off between the probability that secondary infections are more risky than primary ones and reducing the risk of getting a secondary infection. Therefore, results considering risky primary infections may be more relevant. Since third and fourth infections are rarely reported (Fried et al. 2010; Gibbons et al. 2007) it seems reasonable to assume asymptomatic third and fourth infections. We will therefore briefly discuss the optimal vaccination ages we obtained in the case of all four serotypes coexisting when primary infections are considered risky, and third and fourth infections asymptomatic. Assuming a constant efficacy the optimal vaccination age was found to be 10 months. This is, however, significantly below the licensed age range of 9–45 years for Dengvaxia. If vaccination is only possible within this age range, it should be carried out at 108 months which will lead to an increase in lifetime expected risk of roughly 620%. If the vaccine increases the risk in seronegative recipients, the optimal vaccination age was found to be 156 months, i.e. 13 years, and therefore within the permitted age range.

## Electronic supplementary material

Below is the link to the electronic supplementary material.

Supplementary material 1 (pdf 241 KB)
