# Supplementary material for: The Optimal Age of Vaccination Against Dengue with an Age-Dependent Biting Rate with Application to Brazil
Source: Bull Math Biol. 2020 Jan 14;82(1):12. doi: 10.1007/s11538-019-00690-1 (PMC6957571; doi:10.1007/s11538-019-00690-1)
Supplement: Supplementary file 1 — Supplementary material 1 (pdf 241 KB) [file 11538_2019_690_MOESM1_ESM.pdf]

The Optimal Age of Vaccination Against Dengue with an Age-Dependent Biting Rate with Application to Brazil

Sandra B. Maier[1], **Eduardo Massad**[2,4,5], **Marcos Amaku**[2], **Marcelo N. Burattini**[2,3] **and David Greenhalgh**[1],

[1] Department of Mathematics and Statistics, University of Strathclyde, Glasgow G1 1XH, U.K.,

[2] LIM01-Hospital de Clínicas, Faculdade de Medicina, Universidade de São Paulo, São Paulo, SP, Brazil,

[3] Hospital São Paulo, Escola Paulista de Medicina, Universidade Federal de São Paulo, São Paulo, SP, Brazil,

[4] London School of Hygiene and Tropical Medicine, London, U.K.,

[5] School of Applied Mathematics, Fundação Getulio Vargas, Rio de Janeiro, RJ, Brazil.

Supplementary Appendix A. R_0

Supplementary Appendix A.1. Intuitive Derivation of R_0

The basic reproduction number R_0 for the model given in Equations (6) and (7) can be derived by considering how many infections are caused by a single infected human in an otherwise entirely disease-free population at equilibrium similarly to the approach of Massad et al. [1] for their dengue model.

Let $T_{H\to M}(a)$ be the number of infectious mosquitoes that get infectious by biting a single newly infected human who enters an entirely disease-free population at age a. The probability that the individual is still alive and infectious at age $s > a$ is $e^{-\int_a^s(\mu_H(\bar{s})+\gamma_H)d\bar{s}} = \frac{\pi(s)}{\pi(a)}e^{-\gamma_H(s-a)}$.

Using Equation (5) the total cumulative future contribution to the force of infection for mosquitoes is therefore $c\int_a^\infty \frac{q(s)}{N_H}\frac{\pi(s)}{\pi(a)}e^{-\gamma_H(s-a)}ds$. The total number of exposed mosquitoes is then given by $mc\int_a^\infty q(s)\frac{\pi(s)}{\pi(a)}e^{-\gamma_H(s-a)}ds$, and

$$T_{H\to M}(a) = mce^{-\mu_M\tau}\int_a^\infty q(s)\frac{\pi(s)}{\pi(a)}e^{-\gamma_H(s-a)}ds.$$

These infectious mosquitoes will in turn bite susceptible humans and thus cause new infections. Denote the age-distribution of newly infected humans caused by a single mosquito by $T_{M\to H}(a')$. This distribution depends on the density function of susceptible humans $\bar{u}(a')$, the age-dependent biting rate $q(a')$, the age-dependent seroconversion rate $C(a')$, the transmission probability from mosquito to human b, and the expected time a mosquito remains infectious $1/\mu_M$. The density function of unaffected humans is given by $\bar{u}(a') = \frac{N_H}{L}\pi(a')$ since apart from a single human all humans are assumed unaffected. As the mosquito lifetime is short compared with the age-dependent changes in the other quantities we may assume that all the infections occur at approximately age a'. $T_{M\to H}(a')$ is therefore given by

$$T_{M\to H}(a') \approx \frac{b}{\mu_M L} q(a')C(a')\pi(a').$$

Hence $f(a, a')$ the age-distribution of infectious individuals caused by a single newly infected individual of age a entering a disease-free population at equilibrium is

$$f(a, a') = T_{H\to M}(a)T_{M\to H}(a').$$

The basic reproduction number R_0 is given by the spectral radius of this function [2]. As $f(a, a')$ factorises as a function of a multiplied by a function of a' its largest eigenvalue is its trace given by

$$\int_0^\infty f(a, a)da$$

which is Equation (9).

Supplementary Appendix A.2. Approximation of R_0

An approximation of the basic reproduction number given in Equation (9) can be found by linearizing the equations for the density with respect to age of the proportion of infected humans $i_H(a, t) = \frac{I_H(a,t)}{N_H}$ and the proportion of infectious mosquitoes $i_M(t) = \frac{I_M(t)}{N_M}$ at the

beginning of an epidemic. Since the transmission model that we introduce in Section 2 considers the age-distributions in the human population the age-distribution of unaffected at the beginning of an epidemic is given by $U_H(a,t) = \frac{N_H}{L}\pi(a)$ and the linearised solutions $i_H(a,t) = c_H(a)e^{\lambda t}$ and $i_M(t) = c_M e^{\lambda t}$ are considered. Bearing this in mind a similar approach to that of Massad et al. [3] can be followed to get

$$mbce^{-\mu_M \tau} = (\lambda + \mu_M)e^{\lambda\tau} L \left[\int_0^\infty q(a)C(a) \int_a^\infty q(s)e^{-(\gamma_H+\lambda)(s-a)}\pi(s)dsda \right]^{-1}$$

and thus by substituting into Equation (9) the approximation of the basic reproduction number given in Equation (10) is derived.

Supplementary Appendix B. Serostatus-dependent Hospitalisation Risk

In the long-term follow-up of the Dengvaxia trials hospitalisation cases were reported according to serostatus at baseline for individuals in the control group and the vaccine group as shown in Table B.1. These numbers indicate a difference in the risk for vaccinated and unvaccinated seronegative and seropositive individuals. The associated risk functions need to be computed depending on these relative risks of hospitalisation and the vaccine efficacies given in Table 3. However, in this case the assumptions relating to ADE and cross-immunity require more consideration than if we assume that the risk for vaccinated and recovered individuals are the same. In this section we derive the risk functions for risky primary infections in detail, and briefly outline the derivation in the case of ADE implying risk-free primary infections.

Table B.1: Number of hospitalisation cases recorded during the long-term follow-up of clinical phase 3 trials in the vaccine group and control group [4]

		Control Group	Vaccine Group
2-8 years	Seropositive	11/236	9/481
	Seronegative	5/173	17/330
9-16 years	Seropositive	15/752	7/1,546
	Seronegative	4/204	7/382

Supplementary Appendix B.1. Assuming Risky Primary Infections

Consider primary infections to have some associated risk. According to the data presented in Table B.1 the risk of hospitalisation depends on the serostatus, the vaccination history and the age of an individual. We denote the age-classes used in Table B.1 by G_1 and G_2. Further we define the relative risks for individuals of age $a \in G_s$ who are indeed at risk by

$$\bar{h}^-(a) = \frac{P(\text{hospitalisation of unvaccinated seronegative in age-class } G_s)}{P(\text{hospitalisation of unvaccinated seronegative in age-class } G_s)} = 1,$$

$$\bar{h}^+(a) = \frac{P(\text{hospitalisation of unvaccinated seropositive in age-class } G_s)}{P(\text{hospitalisation of unvaccinated seronegative in age-class } G_s)},$$

$$h_*^-(a) = \frac{P(\text{hospitalisation of successfully vaccinated initially seronegative in age-class } G_s)}{P(\text{hospitalisation of unvaccinated seronegative in age-class } G_s)},$$

$$h_*^+(a) = \frac{P(\text{hospitalisation of successfully vaccinated initially seropositive in age-class } G_s)}{P(\text{hospitalisation of unvaccinated seronegative in age-class } G_s)}.$$

Note that all of the relative risks are defined for at risk individuals. In the case of no ADE and no permanent cross-immunity any type of infection is associated with some risk, but only individuals with antibodies to no more than three serotypes are at risk of infection. If two heterologous infections confer permanent cross-immunity only the first two infections are associated with risk. For $h_*^-(a)$ and $h_*^+(a)$ only successfully vaccinated individuals are considered, unsuccessfully vaccinated individuals have the same risks as unvaccinated individuals with the same serostatus. To compute the relative risks the numbers in Table B.1 therefore need to be corrected to reflect the fact that not every individual in the vaccine group was successfully vaccinated and that not all seropositives are at risk. We therefore define the following probabilities for each age-class G_s:

$$p_1(G_s) = P(\text{unvaccinated seropositive is at risk at the pre-vaccination steady state}),$$
$$p_2(G_s) = P(\text{initial seronegative is successfully vaccinated and at risk immediately afterwards}),$$
$$\widetilde{p}_2(G_s) = P(\text{initial seronegative is at risk} \mid \text{successful vaccination against at least one serotype}),$$
$$p_3(G_s) = P(\text{initial seropositive is successfully vaccinated and at risk immediately afterwards}),$$
$$v_e^+(G_s) = P(\text{successful vaccination of an initial seropositive against at least one serotype}),$$
$$v_e^-(G_s) = P(\text{successful vaccination of an initial seronegative against at least one serotype}),$$
$$v_e^i(G_s) = P(\text{successful vaccination of an individual against serotype } i),$$

where $v_e^-(G_s)$, $v_e^+(G_s)$ and $v_e^i(G_s)$ are the serostatus-specific and serotype-specific vaccine efficacies given in Table 3.

Note that $p_2(G_s)$, $\widetilde{p}_2(G_s)$ and $p_3(G_s)$ are evaluated immediately after vaccination. Trivially $p_2(G_s) = \widetilde{p}_2(G_s)v_e^-(G_s)$. However, the computation of $p_1(G_s)$, $\widetilde{p}_2(G_s)$ and $p_3(G_s)$ depends on whether two heterologous infections confer cross-immunity. If there is no cross-immunity the probability $p_1(G_s)$ can be calculated as the probability of being seropositive to at most three serotypes given that an individual is seropositive. The pre-vaccine steady-state fraction of individuals unaffected by serotype i in age-class G_s can be obtained as

$$u_i^0(G_s) = \frac{\frac{N_H}{L}\int_{B_1}^{B_2} u_i^0(a)\pi_H(a)da}{\frac{N_H}{L}\int_{B_1}^{B_2} \pi_H(a)da},$$

where

$$u_i^0(a) = e^{-\int_0^a \lambda_i^0(s)C_i(s)ds}$$

is the pre-vaccine steady-state fraction of individuals unaffected by serotype i at age a and B_1 and B_2 are the limits of the age-class G_s. Therefore

$$p_1(G_s) = \frac{1 - u_1^0(G_s)u_2^0(G_s)u_3^0(G_s)u_4^0(G_s) - \left(1 - u_1^0(G_s)\right)\left(1 - u_2^0(G_s)\right)\left(1 - u_3^0(G_s)\right)\left(1 - u_4^0(G_s)\right)}{1 - u_1^0(G_s)u_2^0(G_s)u_3^0(G_s)u_4^0(G_s)}.$$

In the case of cross-immunity $p_1(G_s)$ is the probability of an individual being seropositive to exactly one serotype given that he or she is seropositive, i.e.

$$p_1(G_s) = \frac{\sum_{i=1}^{4}\left[\left(1 - u_i^0(G_s)\right)u_j^0(G_s)u_k^0(G_s)u_l^0(G_s)\right]}{1 - u_1^0(G_s)u_2^0(G_s)u_3^0(G_s)u_4^0(G_s)}.$$

Similarly for $\widetilde{p}_2(G_s)$ consider the serotype-specific vaccine efficacies $v_e^i(G_s)$ and note that a successfully vaccinated initial seronegative who is at risk in the case of no cross-immunity

has been vaccinated against at least one and no more than three serotypes, i.e.

$$\widetilde{p}_2(G_s) = \frac{1 - v_e^1(G_s)v_e^2(G_s)v_e^3(G_s)v_e^4(G_s) - \left(1 - v_e^1(G_s)\right)\left(1 - v_e^2(G_s)\right)\left(1 - v_e^3(G_s)\right)\left(1 - v_e^4(G_s)\right)}{1 - (1 - v_e^1(G_s))(1 - v_e^2(G_s))(1 - v_e^3(G_s))(1 - v_e^4(G_s))}.$$

In the case of cross-immunity individuals who have been vaccinated against exactly one serotype need to be considered, i.e.

$$\widetilde{p}_2(G_s) = \frac{\sum_{i=1}^{4}\left[v_e^i(G_s)\left(1 - v_e^j(G_s)\right)\left(1 - v_e^k(G_s)\right)\left(1 - v_e^l(G_s)\right)\right]}{1 - (1 - v_e^1(G_s))(1 - v_e^2(G_s))(1 - v_e^3(G_s))(1 - v_e^4(G_s))}.$$

In the case of permanent cross-immunity after two heterologous infections clearly $p_3(G_s) = 0$ and by definition $h_*^+(a) = 0$ since no initially seropositive individual who was successfully vaccinated is at risk. However, if there is no cross-immunity individuals who are seropositive to at most three serotypes after vaccination remain at risk. From the steady-state analysis we know that the distributions of seropositivity for individuals in age-class G_S to serotype i only, and serotypes i and j only, conditional on seropositivity are given by

$$s_i(G_s) = \frac{\left(1 - u_i^0(G_s)\right)u_j^0(G_s)u_k^0(G_s)u_l^0(G_s)}{1 - u_i^0(G_s)u_j^0(G_s)u_k^0(G_s)u_l^0(G_s)}, \text{ and}$$
$$s_{ij}(G_s) = \frac{\left(1 - u_i^0(G_s)\right)\left(1 - u_j^0(G_s)\right)u_k^0(G_s)u_l^0(G_s)}{1 - u_i^0(G_s)u_j^0(G_s)u_k^0(G_s)u_l^0(G_s)}$$

respectively. An individual who was initially seropositive to serotype i and then successfully vaccinated remains at risk if the vaccine was successful against no more than two other serotypes. Similarly an individual who was seropositive to serotypes i and j remains at risk after successful vaccination against only one more serotype. The probabilities of this happening in age-class G_s are given by

$$p_r^i(G_s) = s_i(G_s)v_e^+(G_s)\frac{1 - v_e^j(G_s)v_e^k(G_s)v_e^l(G_s) - \left(1 - v_e^j(G_s)\right)\left(1 - v_e^k(G_s)\right)\left(1 - v_e^l(G_s)\right)}{1 - (1 - v_e^j(G_s))(1 - v_e^k(G_s))(1 - v_e^l(G_s))}, \text{ and}$$
$$p_r^{ij}(G_s) = s_{ij}(G_s)v_e^+(G_s)\frac{1 - v_e^k(G_s)v_e^l(G_s) - \left(1 - v_e^k(G_s)\right)\left(1 - v_e^l(G_s)\right)}{1 - (1 - v_e^k(G_s))(1 - v_e^l(G_s))}$$

respectively for the two cases. Individuals who were initially seropositive to three serotypes

are no longer at risk after successful vaccination and those initially seropositive to all serotypes cannot be vaccinated. The probability $p_3(G_s)$ in the case of no cross-immunity is therefore given by

$$p_3(G_s) = \sum_{i=1}^{4} p_r^i(G_s) + \sum_{\substack{i,j=1 \\ i\neq j}}^{4} p_r^{ij}(G_s).$$

We have

$$\bar{h}^-(a) = 1$$

by definition. Additionally using Table B.1 we have

$$\bar{h}^+(a) = \begin{cases} \frac{11}{236p_1(G_1)} \Big/ \frac{5}{173}, & 0 \leq a < 9, \\ \frac{15}{752p_1(G_2)} \Big/ \frac{4}{204}, & 9 \leq a < \infty, \end{cases}$$

since for example of the 236 initial seropositives aged 2-8 in the control group only $236p_1(G_1)$ were at risk. Similarly only $481p_3(G_1)$ individuals were at risk vaccinated seropositive individuals aged 2-8 years immediately after vaccination, and therefore

$$h_*^+(a) = \begin{cases} \frac{9}{481p_3(G_1)} \Big/ \frac{11}{236p_1(G_1)}, & 0 \leq a < 9, \\ \frac{7}{1{,}546p_3(G_2)} \Big/ \frac{15}{752p_1(G_2)}, & 9 \leq a < \infty. \end{cases}$$

To find the relative risk $h_*^-(a)$ for the age-group 2-8 years note that out of the 330 initially seronegative individuals in the vaccine group $330v_e^-(G_1)\widetilde{p}_2(G_1) = 330p_2(G_1)$ were successfully vaccinated and remain at risk, while $330v_e^-(G_1)(1-\widetilde{p}_2(G_1))$ were successfully vaccinated and are no longer at risk, and $330(1 - v_e^-(G_1))$ were not successfully vaccinated. The number of hospitalisation cases in unsuccessfully vaccinated individuals is therefore $330(1 - v_e^-(G_1))\frac{5}{173}$ with the remaining $17 - 330(1 - v_e^-(G_1))\frac{5}{173}$ hospitalisations having occurred in at risk successfully vaccinated initially seronegative individuals. For the age-group 9-16 years a similar argument can be followed and the relative risk for successfully vaccinated initially seronega-

tives is therefore given by

$$h_*^-(a) = \begin{cases} \frac{17-330(1-v_e^-(G_1))\frac{5}{173}}{330p_2(G_1)} \Big/ \frac{5}{173}, & 0 \leq a < 9, \\ \frac{7-382(1-v_e^-(G_2))\frac{4}{204}}{382p_2(G_2)} \Big/ \frac{4}{204}, & 9 \leq a < \infty. \end{cases}$$

Having found the relative risks $\bar{h}^-(a)$, $\bar{h}^+(a)$, $h_*^-(a)$ and $h_*^+(a)$ the pre-vaccine hospitalisation risk $R(a)$ given in Equation (19) can now be used to determine the risk functions associated with any type of infection. Define $R^-(a)$ to be the pre-vaccination hospitalisation risk for seronegative individuals and $R^+(a)$ to be the pre-vaccination hospitalisation risk for at risk seropositive individuals. Note that $R^+(a) = \bar{h}^+(a)R^-(a)$. Then in the case of no cross-immunity we have

$$\begin{aligned} R(a) = u_1^0(a)u_2^0(a)u_3^0(a)u_4^0(a)R^-(a) + \big[1 - u_1^0(a)u_2^0(a)u_3^0(a)u_4^0(a) \\ - \big(1-u_1^0(a)\big)\big(1-u_2^0(a)\big)\big(1-u_3^0(a)\big)\big(1-u_4^0(a)\big)\big] R^+(a), \\ = \big\{u_1^0(a)u_2^0(a)\, u_3^0(a)u_4^0(a) + \big[1 - u_1^0(a)u_2^0(a)u_3^0(a)u_4^0(a) \\ - \big(1-u_1^0(a)\big)\big(1-u_2^0(a)\big)\big(1-u_3^0(a)\big)\big(1-u_4^0(a)\big)\big] \bar{h}^+(a)\big\} R^-(a), \end{aligned}$$

and

$$\begin{aligned} &R_{\bar{i}\bar{j}\bar{k}\bar{l}}(a) = \bar{h}^-(a)R^-(a) = R^-(a), \\ &R_{ijkl}(a) = R_{ijk\bar{l}}(a) = R_{ij\bar{k}\bar{l}}(a) = \bar{h}^+(a)R^-(a), \\ &R_{ij_*k_*l_*}(a) = R_{ij_*k_*\bar{l}}(a) = R_{ij_*\bar{k}\bar{l}}(a) = h_*^-(a)R^-(a), \\ &R_{ijkl_*}(a) = R_{ijk_*l_*}(a) = R_{ijk_*\bar{l}}(a) = h_*^+(a)R^+(a) = h_*^+(a)\bar{h}^+(a)R^-(a). \end{aligned}$$

Note that for vaccinated individuals who have experienced a natural infection at some point we assume the risk to be that of successfully vaccinated seropositive individuals who were seropositive at baseline independent of when the natural infection occurred. In the case of cross-immunity only seropositives who are seropositive to exactly one serotype experience a

risk, i.e. in this case

$$
\begin{aligned}
R(a) &= u_1^0(a)u_2^0(a)u_3^0(a)u_4^0(a)R^-(a) + \sum_{i=1}^{4} \left[(1-u_i^0(a))u_j^0(a)u_k^0(a)u_l^0(a)\right] R^+(a), \\
&= \left\{ u_1^0(a)u_2^0(a)u_3^0(a)u_4^0(a) + \sum_{i=1}^{4} \left[(1-u_i^0(a))u_j^0(a)u_k^0(a)u_l^0(a)\right] \bar{h}^+(a) \right\} R^-(a),
\end{aligned}
$$

and the risk functions are

$$
\begin{aligned}
&R_{\bar{i}\bar{j}\bar{k}\bar{l}}(a) = \bar{h}^-(a)R^-(a) = R^-(a), \\
&R_{i\bar{j}\bar{k}\bar{l}}(a) = \bar{h}^+(a)R^-(a), \\
&R_{ij_*\bar{k}\bar{l}}(a) = h_*^-(a)R^-(a), \\
&R_{ijkl}(a) = R_{ijk\bar{l}}(a) = R_{ij_*k_*l_*}(a) = R_{ij_*k_*\bar{l}}(a) = R_{ijkl_*}(a) = R_{ijk_*l_*}(a) = R_{ijk_*\bar{l}}(a) = 0.
\end{aligned}
$$

Supplementary Appendix B.2. Assuming Risk-free Primary Infections

Assuming ADE to imply that all primary infections are risk-free requires a redefinition of $\bar{h}^-(a)$, $\bar{h}^+(a)$, and $h_*^-(a)$ relative to unvaccinated seropositives instead of unvaccinated seronegatives. In this case $\bar{h}^-(a) = 0$ and $\bar{h}^+(a) = 1$. The definition of $h_*^+(a)$ is unchanged and this relative risk can be computed as above. $h_*^-(a)$ can be derived similarly to when primary infections are risky, however only at risk seropositives are considered and therefore

$$
h_*^-(a) = \begin{cases} \frac{17-330(1-v_e^-(G_1))\frac{5}{173}}{330p_2(G_1)} \Big/ \frac{11}{236p_1(G_1)}, & 0 \le a < 9, \\ \frac{7-382(1-v_e^-(G_2))\frac{4}{204}}{382p_2(G_2)} \Big/ \frac{15}{752p_1(G_2)}, & 9 \le a < \infty. \end{cases}
$$

The risks associated with any type of infection now need to be expressed in terms of $R^+(a)$ since we assume that $R^-(a) = 0$.

If there is no permanent cross-immunity after two heterologous infections we have

$$
R(a) = \left[1 - u_1^0(a)u_2^0(a)u_3^0(a)u_4^0(a) - \left(1-u_1^0(a)\right)\left(1-u_2^0(a)\right)\left(1-u_3^0(a)\right)\left(1-u_4^0(a)\right)\right] R^+(a),
$$

and

$$R_{i\bar{j}\bar{k}\bar{l}}(a) = 0,$$
$$R_{ijkl}(a) = R_{ijk\bar{l}}(a) = R_{ij\bar{k}\bar{l}}(a) = \bar{h}^{+}(a)R^{+}(a) = R^{+}(a),$$
$$R_{ij_*k_*l_*}(a) = R_{ij_*k_*\bar{l}}(a) = R_{ij_*\bar{k}\bar{l}}(a) = h_*^{-}(a)R^{+}(a),$$
$$R_{ijkl_*}(a) = R_{ijk_*l_*}(a) = R_{ijk_*\bar{l}}(a) = h_*^{+}(a)R^{+}(a).$$

If there is permanent cross-immunity after two heterologous infections we have

$$R(a) = \sum_{i=1}^{4} \left[(1 - u_i^0(a))u_j^0(a)u_k^0(a)u_l^0(a)\right] R^{+}(a),$$

and

$$R_{ij\bar{k}\bar{l}}(a) = \bar{h}^{+}(a)R^{+}(a) = R^{+}(a),$$
$$R_{ij_*\bar{k}\bar{l}}(a) = h_*^{-}(a)R^{+}(a),$$
$$R_{ijkl}(a) = R_{ijk\bar{l}}(a) = R_{i\bar{j}\bar{k}\bar{l}}(a) = 0,$$
$$R_{ij_*k_*l_*}(a) = R_{ij_*k_*\bar{l}}(a) = R_{ijkl_*}(a) = R_{ijk_*l_*}(a) = R_{ijk_*\bar{l}}(a) = 0.$$

References

[1] E. Massad, F. Coutinho, M. Burattini, M. Amaku, Estimation of R_0 from the initial phase of an outbreak of a vector-borne infection, Tropical Medicine and International Health 15 (2010) 120–126.

[2] O. Diekmann, J. A. P. Heesterbeek, J. A. Metz, On the definition and the computation of the basic reproduction ratio R_0 in models for infectious diseases in heterogeneous populations, Journal of Mathematical Biology 28 (1990) 365–382.

[3] E. Massad, F. A. B. Coutinho, M. N. Burattini, L. F. Lopez, The risk of yellow fever in a dengue-infested area, Transactions of the Royal Society of Tropical Medicine and Hygiene 95 (2001) 370–374.

[4] SAGE/World Health Organization, Background paper on dengue vaccines, 2016. http://www.who.int/immunization/sage/meetings/2016/april/presentations_background_docs/en/ (last accessed: May 3 2018).
